# A Particle Swarm Optimization-Guided Ivy Algorithm for Global Optimization Problems

**DOI:** 10.3390/biomimetics10050342

**Published:** 2025-05-21

**Authors:** Kaifan Zhang, Fujiang Yuan, Yang Jiang, Zebing Mao, Zihao Zuo, Yanhong Peng

**Affiliations:** 1 School of Computer Science, Hubei University of Technology, Wuhan 430068, China; 2 School of Computer Science and Technology, Taiyuan Normal University, Taiyuan 030619, China; 3 College of Mechanical Engineering, Chongqing University of Technology, Chongqing 400054, China; 4 Department of Engineering Science and Mechanics, Shibaura Institute of Technology, 3-7-5 Toyosu, Koto-ku, Tokyo 135-8548, Japan; 5 Chongqing Institute of Green and Intelligent Technology, Chinese Academy of Sciences, Chongqing 400714, China

**Keywords:** particle swarm optimization, ivy algorithm, global optimization ability

## Abstract

In recent years, metaheuristic algorithms have garnered significant attention for their efficiency in solving complex optimization problems. However, their performance critically depends on maintaining a balance between global exploration and local exploitation; a deficiency in either can result in premature convergence to local optima or low convergence efficiency. To address this challenge, this paper proposes an enhanced ivy algorithm guided by a particle swarm optimization (PSO) mechanism, referred to as IVYPSO. This hybrid approach integrates PSO’s velocity update strategy for global searches with the ivy algorithm’s growth strategy for local exploitation and introduces an ivy-inspired variable to intensify random perturbations. These enhancements collectively improve the algorithm’s ability to escape local optima and enhance the search stability. Furthermore, IVYPSO adaptively selects between local growth and global diffusion strategies based on the fitness difference between the current solution and the global best, thereby improving the solution diversity and convergence accuracy. To assess the effectiveness of IVYPSO, comprehensive experiments were conducted on 26 standard benchmark functions and three real-world engineering optimization problems, with the performance compared against 11 state-of-the-art intelligent optimization algorithms. The results demonstrate that IVYPSO outperformed most competing algorithms on the majority of benchmark functions, exhibiting superior search capability and robustness. In the stability analysis, IVYPSO consistently achieved the global optimum across multiple runs on the three engineering cases with reduced computational time, attaining a 100% success rate (SR), which highlights its strong global optimization ability and excellent repeatability.

## 1. Introduction

Metaheuristic algorithms have become vital tools for addressing complex optimization problems and are widely applied in fields such as engineering design [1,2], machine learning [3,4], and control systems [5,6]. Compared to traditional mathematical optimization techniques, such as gradient-based techniques [7], metaheuristics offer several advantages; they do not require gradient information, exhibit low sensitivity to initial conditions, and are well-suited for high-dimensional or multimodal problems [8,9]. Their flexibility in navigating the solution space and capability to avoid entrapment in local optima have made them a central focus in computational intelligence research [10,11].

In recent years, hybridization techniques have been increasingly employed to enhance the performance of metaheuristics by integrating complementary strategies from different algorithms, thereby improving the convergence speed, solution quality, and robustness [12,13,14,15]. Among these, particle swarm optimization (PSO) has received considerable attention due to its simplicity and rapid convergence [16]. However, PSO also suffers from limited local exploitation ability and a tendency toward premature convergence. To mitigate these issues, numerous studies have combined particle swarm optimization (PSO) with evolutionary operators such as genetic algorithms (GA) [17] and differential evolution (DE) [18], adaptive parameter control techniques [19], and multi-strategy perturbation schemes to better balance global exploration and local exploitation [20].

In parallel, the ivy algorithm (IVYA) has emerged as a promising bio-inspired approach due to its straightforward design and strong local search capabilities [21]. By mimicking the natural growth and propagation behaviors of ivy plants, IVYA can efficiently exploit the neighborhood of promising regions while preserving population diversity. Nevertheless, the lack of a global guidance mechanism limits its convergence efficiency on large-scale optimization problems.

Despite the development of various hybrid metaheuristics, most existing methods primarily focus on accelerating convergence or maintaining diversity, without effectively addressing how to preserve the exploration–exploitation balance while avoiding premature convergence. Moreover, mechanisms that adaptively adjust search strategies based on dynamic fitness landscapes remain underexplored in the current literature.

To bridge these gaps, this paper introduces a novel hybrid optimization algorithm—IVYPSO—which synergistically combines the global guidance of PSO with the adaptive local search capabilities of IVYA. IVYPSO incorporates a biologically inspired ivy perturbation variable (GV) into the PSO velocity update equation, enabling adaptive directional control and stochastic perturbations based on the current fitness landscape. This design enhances the algorithm’s ability to escape local optima while improving the population diversity and convergence stability. The main contributions of this work are summarized as follows:

1. A novel PSO-guided hybrid optimization algorithm, IVYPSO, is proposed. It embeds the ivy growth strategy of IVYA into the velocity update process of PSO, thereby enabling dynamic switching between local and global search modes.
2. A balanced search framework was constructed by comparing the fitness of the current individual with that of the global best. The algorithm dynamically determines whether to perform local exploitation or global exploration, thereby improving its adaptability to complex search spaces.
3. A comprehensive experimental evaluation was conducted on 26 standard benchmark functions and three constrained real-world engineering design problems. The performance of the proposed IVYPSO algorithm was compared against eleven advanced metaheuristic optimization algorithms, including classical methods (such as PSO, IVY, BOA, WOA, and GOOSE), as well as recently developed hybrid algorithms from high-quality research studies (including HPSOBOA, FDC-AGDE, dFDB-LSHADE, NSM-BO, dFDB-SFS, and FDB-AGSK). The experimental results demonstrate that IVYPSO consistently outperformed the compared algorithms in terms of optimization accuracy, convergence speed, and robustness.

The rest of this paper is organized as follows. Section 2 reviews the related work concerning the design and development of metaheuristic search algorithms. Section 3 introduces the PSO and ivy algorithms. Section 4 presents the proposed IVYPSO hybrid algorithm. Section 5 covers its testing on benchmark functions and applications for practical engineering problems. Section 6 discusses the results and outlines future work.

## 2. Related Work

Metaheuristic algorithms have become essential tools for addressing complex, multimodal, and high-dimensional optimization problems. Inspired by natural, biological, or social phenomena, these gradient-free algorithms are particularly well-suited for real-world engineering applications. Recent advancements in metaheuristics have shifted the research focus toward enhancing the algorithmic performance by redesigning key components—namely, the guiding mechanisms, convergence strategies, and update schemes.

Among these components, the guiding mechanism is critical, as it governs how individuals or candidate solutions are influenced by others within the population. Traditional guidance approaches, such as those used in classical PSO, typically direct individuals toward global or local optima based on fitness values but often suffer from premature convergence and loss of population diversity. To address these challenges, advanced guiding strategies have been developed, including fitness distance balance (FDB) [22], adaptive FDB (AFDB) [23], dynamic FDB (DFDB) [24], and fitness distance constraint (FDC). By incorporating spatial distance information between solutions, these methods achieve a more balanced trade-off between exploration and exploitation, as demonstrated by their successful integration into algorithms such as FDC_AGDE [25], dFDB_LSHADE [26], dFDB_SFS [27], and FDB_AGSK [28], which exhibit strong performance on benchmark functions and engineering problems.

Convergence strategies, which determine how candidate solutions are updated each iteration, also play a pivotal role in directing search trajectories and influencing the convergence speed. The contemporary approaches include time-decreasing control parameters, multi-phase convergence schemes, and hybrid deterministic random update models. For instance, the GOOSE algorithm adapts its convergence behavior across different search stages [29], while HPSOBOA incorporates multiple convergence modes to sustain robustness across diverse problem domains [30].

Moreover, update schemes—particularly for the selection of surviving individuals—are vital for maintaining the solution quality and population diversity. Conventional methods relying solely on fitness-based ranking risk stagnation and premature convergence. To overcome this, the natural survivor method (NSM) integrates the fitness performance with historical success rates to judiciously select individuals for the next generation [31]. NSM has proven effective in algorithms such as NSM-BO and NSM-LSHADE-CnEpSin [32], markedly enhancing the stability and global search performance in constrained engineering problems.

Despite these advances, most existing studies focus on improving individual algorithmic components—such as the convergence behavior or update strategies—often at the expense of overall adaptability or diversity. A unified framework that simultaneously integrates global guidance and bio-inspired local searches with dynamic adaptability remains lacking.

To bridge this gap, we propose a hybrid algorithm, IVYPSO, which synergistically combines PSO’s global search capability with the ivy algorithm’s local growth and diffusion mechanisms. By embedding a dynamic ivy-inspired variable GV within the PSO velocity update formula, IVYPSO adaptively switches search strategies based on individual fitness, effectively balancing exploration and exploitation. This design not only preserves the convergence accuracy but also enhances the robustness and diversity, offering a novel high-performance optimization framework tailored for complex global optimization challenges.

## 3. Materials and Methods

To better illustrate our hybrid algorithm, the inspiration behind and detailed implementation of the PSO algorithm and ivy algorithm will be introduced.

### 3.1. PSO Formulation

PSO is a nature-inspired swarm intelligence algorithm, proposed by Kennedy and Eberhart in 1995. Its core idea is based on simulating the foraging behavior of bird flocks or fish schools. By sharing information and collaborating among individuals, PSO strikes a balance between global exploration and local exploitation, effectively solving optimization problems.

In the PSO algorithm, each particle has a unique position $X_{i}=[x_{i1},x_{i2},x_{i3},\ldots ,x_{ij},\ldots ,x_{iD}]$ and velocity $V_{i}=\left[\begin{array}{c} v_{i1},v_{i2},v_{i3},\ldots ,v_{ij},\ldots ,v_{iD} \end{array}\right]$, representing a potential solution. Here, j=1,2,…,D, D denotes the dimensionality of the search space. In each iteration, the position of the particle is dynamically updated based on the following three components:

(1) Inertia component: Determined by the particle’s previous velocity, it is used to maintain the particle’s movement trend and balance the global exploration capability of the search.
(2) Individual cognitive component: The particle adjusts its search direction based on its own historical best position $P_{best}$, simulating individual learning behavior.
(3) Social component: The particle adjusts its direction based on the entire population’s global best position $G_{best}$, reflecting social learning and collaborative effects.

During the search process, the particle’s position is influenced by its best position in the neighborhood $P_{best,i}$ and the global best position $G_{best}$ of the entire population.

The particle position and velocity update formulas are given by Equations (1) and (2), respectively:

(1) $$X_{i}^{k+1}=X_{i}^{k}+V_{i}^{k+1}$$

(2) $$V_{i}^{k+1}=wV_{i}^{k}+c_{1}r_{1}\left(P_{best,i}-X_{i}^{k}\right)+c_{2}r_{2}\left(G_{best}-X_{i}^{k}\right)$$

where w is the inertia weight, which controls the balance between global and local searches; $c_{1}$ and $c_{2}$ are acceleration coefficients that determine the influence of individual and group learning, respectively; $c_{1}r_{1}$ and $c_{2}r_{2}$ are random numbers, ranging from 0 to 1, which enhance the randomness of the search.

**Figure 1:**
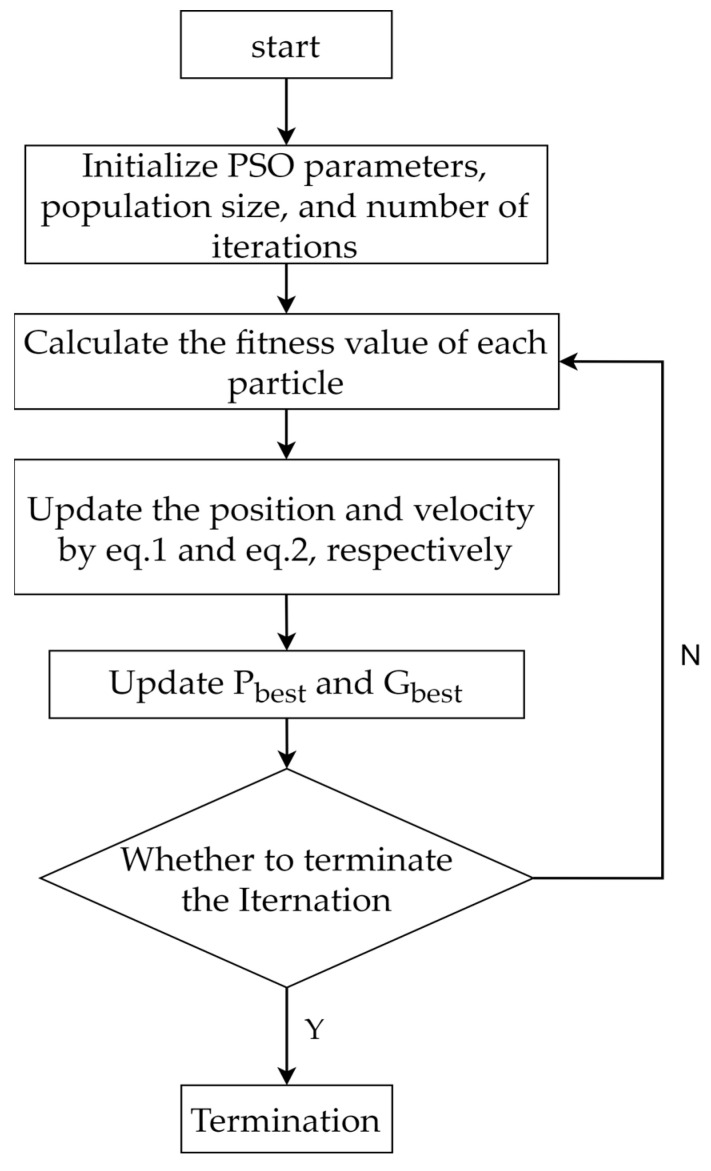
Flowchart of PSO algorithm.

The flowchart of the PSO algorithm is shown in Figure 1.

### 3.2. IVYA Formulation

**Figure 2:**
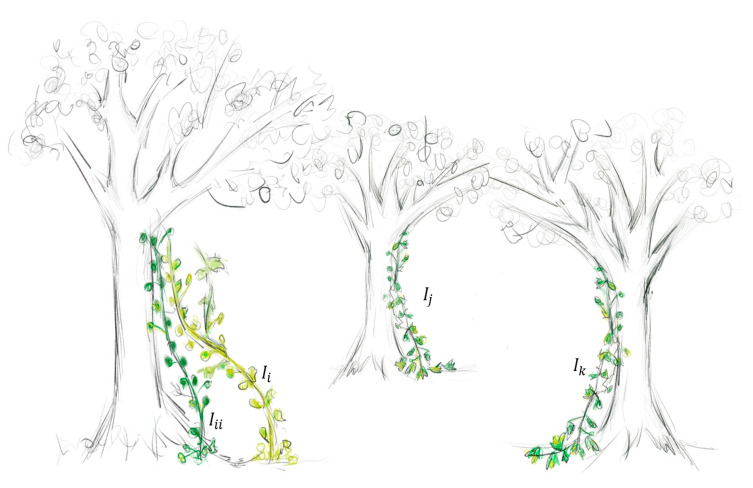
The i−th member of the population $I_{i}$ chooses its closest, most vital neighbor $I_{\mathrm{ii}}$.

**Figure 3:**
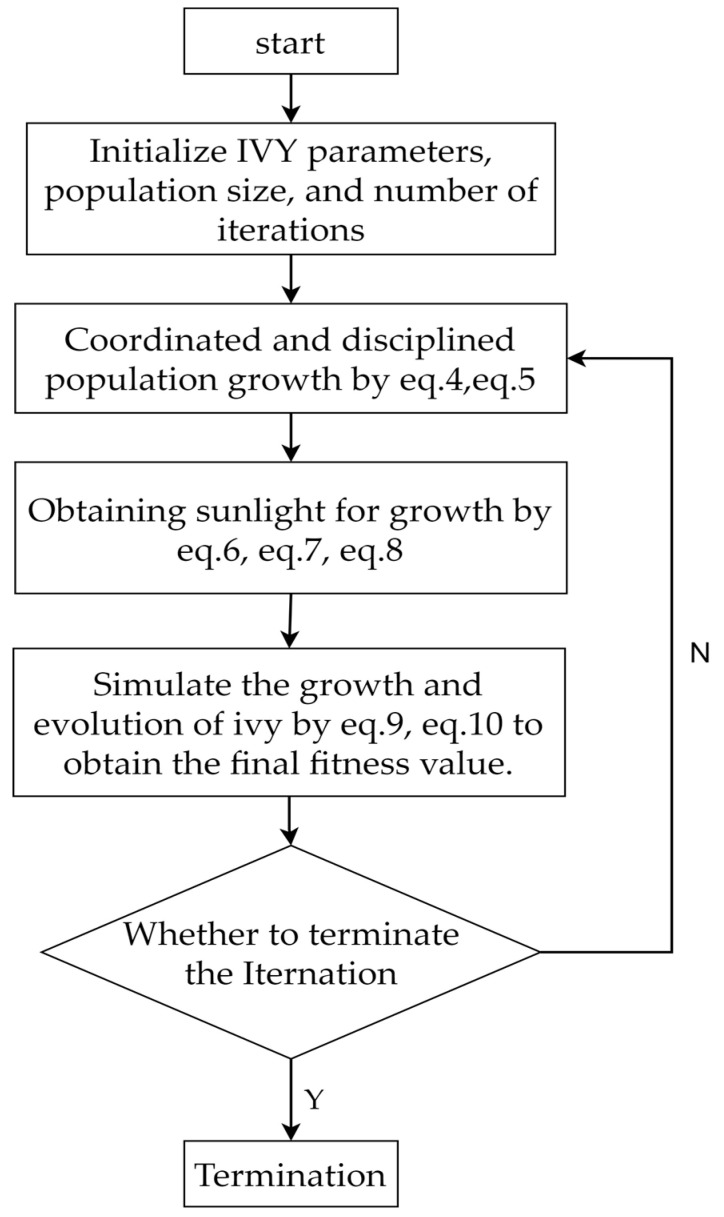
Flowchart of the ivy algorithm.

The ivy algorithm, derived from the growth behavior of ivy plants in nature, is a swarm intelligence optimization method. Ivy plants continuously grow, climb, and spread in the environment in search of sunlight, nutrients, and other resources for survival. This process serves as inspiration for addressing global optimization problems. The algorithm simulates the different life stages of ivy, including growth, ascent, and spreading [33]. The algorithm’s implementation process can be outlined in the subsequent four steps:

(1) Initialize the population, where N and D represent the total number of members and the dimensionality of the problem, respectively. Thus, the i−th population member has the form $I_{i}=(I_{i1},\ldots ,I_{iD})$, where i=1,2,…,N, The total population of ivy plants is represented as $\vec{I}=(I_{1},\ldots ,I_{i},\ldots ,I_{Npop})$, At the start of the algorithm, the initial positions of the ivy algorithm population in the search space are determined using Equation (3):
  (3) $$I_{i}=I_{min}+rand(1,D)⊙(I_{max}-I_{min}),i=1,\ldots ,N$$
  where rand(1,D) represents a vector of dimension D with random numbers uniformly distributed in the range [0, 1]. The upper and lower limits of the search space are denoted by $I_{max}$ and $I_{min}$, respectively, and ⊙ denotes the element-wise product of two vectors.
(2) Coordinated and disciplined population growth. In the growth process of the ivy algorithm, we assume that the growth rate GV of the ivy algorithm is a function of time, expressed through a differential equation, as illustrated in Equation (4):
  (4) $$\frac{dGV(t)}{dt}=\psi \cdot GV(t)\cdot \varphi (GV(t))$$
  where GV, ψ, and φ represent the growth rate, growth velocity, and correction factor for growth deviation, respectively. The member $I_{i}$ is modeled by Equation (5).
  (5) $$\Delta {GV}_{i}(t+1)={rand}^{2}⊙(N(1,D)⊙\Delta {GV}_{i}(t))$$
  where ${GV}_{i}(t)$ and ${GV}_{i}(t+1)$ represent the growth rates at discrete time steps t and t+1, respectively; rand is a random number in the range of [0, 1]; N(1,D) represents a random vector of dimension D.
(3) Obtaining sunlight for growth. For ivy in nature, quickly finding a surface to attach to is crucial. The movement towards the light source is modeled by Equations (6)–(8). In the proposed algorithm, this behavior is simulated by the i−th individual $I_{i}$ in the population, selecting its closest and most optimal neighbor $I_{ii}$ (based on the value of the fitness function) as a reference for self-improvement, as shown in Figure 2.
  (6) $$I_{ii}=\left\{\begin{array}{c} I_{j-1}^{s},I_{i}=I_{j}^{s}\\ I_{i},I_{i}=I_{best} \end{array}\right.$$
  (7) $$I_{i}^{new1}=I_{i}+\left|N(1,D)\right|⊙(I_{ii}-I_{i})+N(1,D)⊙\Delta {GV}_{i},i=1,2,\ldots ,N$$
  (8) $$\left.\Delta Gv_{i}=\left\{\begin{array}{c} I_{i}⊗(I_{max}-I_{min}),Iter=1\\ {rand}^{2}⊙(N(1,D)⊙\Delta Gv_{i}),Iter>1 \end{array}\right.\right.$$
  where $\left|N(1,D)\right|$ is a vector, with each component being the absolute value of the corresponding component in the vector N(1,D).
(4) Growth and evolution of ivy. After the member $I_{i}$ navigates the search space globally to reach its nearest and most significant neighbor $I_{\mathrm{ii}}$, it enters a phase where $I_{i}$ strives to directly follow the optimal member $I_{best}$ in the population. This stage aligns with the pursuit of an improved optimal solution in the vicinity of $I_{best}$, as depicted in Equations (9) and (10).
  (9) $$I_{i}^{new}=I_{Best}⊙(rand(1,D)+N(1,D)⊙\Delta {GV}_{i})$$
  (10) $$\Delta {GV}_{i}^{new}=I_{i}^{new}⊗(I_{max}-I_{min})$$
  The flowchart of the ivy algorithm is shown in Figure 3.

## 4. Proposed Optimization Formulation of IVYPSO

The IVYPSO hybrid algorithm integrates the global exploration capability of PSO with the local exploitation and adaptive perturbation mechanism inspired by the natural growth behavior of ivy plants. Specifically, the algorithm leverages the PSO’s velocity-position update rule to guide individuals toward promising areas in the solution space. Meanwhile, an ivy-inspired GV introduces fine-grained random perturbations to enhance the local search capability around high-quality solutions. To balance exploration and exploitation, IVYPSO adaptively switches between global and local strategies based on a dynamic quality threshold. A greedy selection strategy is also employed to retain elite solutions in each iteration. This coordinated hybridization approach improves the convergence speed, avoids local optima, and enhances the solution’s overall accuracy.

### 4.1. Initialization Phase

In this phase, the objective function fobj is defined to calculate the fitness. The lower and upper bounds of the search space, lb and ub, respectively, are set to restrict the solution range; N individuals are randomly generated, and the position and velocity of each individual i are initialized, along with the ivy variables. The specific parameter initialization process is as follows.

The particles’ initial positions in the search space are randomly generated, as described in Equation (11):

(11) $$X_{i}=unifrnd\left(lb,ub,\left[1,dim\right]\right).$$

The velocity is initialized as a zero vector, as described in Equation (12):

(12) $$V_{i}=zeros\left(1,dim\right).$$

The ivy growth variable GV is then initialized, as described in Equation (13):

(13) $$GV=\frac{X_{i}}{ub-lb}$$

The ivy variable GV represents the relative growth behavior of an ivy plant within the bounded search space and is used to control the intensity of local random movement.

### 4.2. Guidance Mechanism: PSO-Guided Velocity Update

This step updates the velocity for each individual, as described by the velocity update formula in Equation (14):

(14) $$V_{i}^{t+1}=w\cdot V_{i}^{t}+c_{1}\cdot r_{1}\cdot (P_{best}-X_{i}^{t})+c_{2}\cdot r_{2}\cdot (G_{best}-X_{i}^{t})$$

where w=0.7, $c_{1}=1.5$, $c_{2}=1.5$, $r_{1}$, and $r_{2}$ are randomly generated values within the interval [0, 1], adding randomness to the particles. This mechanism guides particles towards their personal best position $P_{best}$ and the global best position $G_{best}$, promoting convergence toward high-quality areas.

This velocity update represents the guidance mechanism in a metaheuristic search (MHS), guiding particles toward personal and global best positions to balance exploration and exploitation. Recent state-of-the-art (SOTA) methods such as FDB and its variants improve the guidance by considering both the fitness and distance to avoid premature convergence.

### 4.3. Update Mechanism: Position Update with Ivy Perturbation

To achieve a dynamic balance between global and local searches, we introduce a dynamic control factor $\beta_{1}$, which is a dynamic adjustment parameter that controls the ratio between global and local search. The expression is as described in Equation (15):

(15) $$\beta_{1}=1+\frac{rand}{2}$$

The random value rand is in the range of [0, 1], and the value of $\beta_{1}$ dynamically changes, which helps introduce randomness and balance the strengths of global and local searches. By adjusting $\beta_{1}$, the algorithm primarily focuses on global searches in the early stages and gradually enhances the local search capability in the later stages, thereby improving the convergence accuracy.

In each iteration, for each individual I the update strategy is selected based on the relationship between its current fitness value $Cost(X_{i})$ and the global best fitness value $Cost(G_{best})$. The condition judgment formula is as described in Equation (16):

(16) $$Cost(X_{i})<\beta_{1}\cdot Cost(G_{best})$$

where $Cost(X_{i})$ represents the fitness value of the current individual and $Cost(G_{best})$ represents the fitness value of the current global best solution. If the condition is met, Equation (17) is used for local searches (exploited around a neighbor). Otherwise, Equation (18) is applied to global searches (perturbed toward the best). Equations (17) and (18) are as follows:

(17) $$X_{i}^{\prime }=x+|randn|\times (x_{neighbor}-x)+randn\times GV$$

(18) $$X_{i}^{\prime }=G_{best}\times (rand+randn\times GV)$$

Here, GV controls the perturbation magnitude; randn is a normally distributed random value that mimics the irregular yet directed growth of ivy tips toward better areas. The ivy growth variable is then updated using Equation (19).

(19) $$GV=GV\times (rand^{2}\times randn)$$

This adaptive update allows GV to decay or intensify based on the stochastic process, simulating flexible growth behaviors for refining solutions.

### 4.4. Survivor Selection Strategy

In metaheuristic search algorithms, survivor selection is a crucial component of the update mechanism that determines which individuals are retained in the population to balance the convergence speed and solution diversity. In IVYPSO, we employ a greedy selection strategy, described in Equation (20), to preserve improved solutions:

(20) $$Cost(X_{i}^{\prime })<Cost(X_{i})⟹X_{i}(t+1)=X_{i}^{\prime }$$

If the new position $X_{i}^{\prime }$ yields a better fitness than the current position $X_{i}$, it replaces the current solution; otherwise, the original solution is retained. Additionally, the individual best $P_{best,i}$ and global best $G_{best}$ are updated synchronously upon improvement.

Furthermore, inspired by recent advances in metaheuristics, the NSM has been proposed as an effective survivor selection technique. NSM dynamically combines fitness and historical success information to select survivors, enhancing the stability and diversity in the population. Although IVYPSO does not explicitly implement NSM, its greedy selection strategy combined with ivy-inspired perturbations shares conceptual similarities with NSM’s goal of balancing exploitation and exploration, ensuring robustness against premature convergence. Future work may explore integrating NSM directly into IVYPSO to further improve the update mechanism performance.

### 4.5. Summary

**Figure 4:**
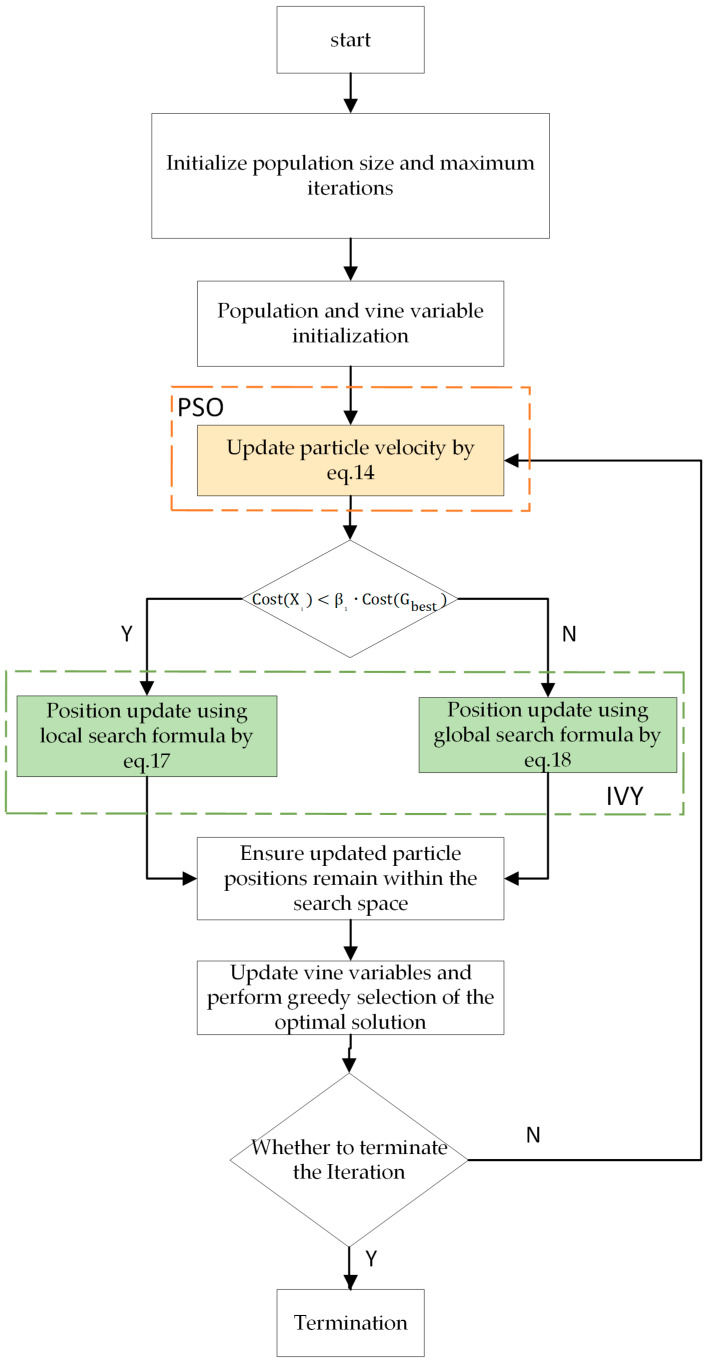
Flowchart of IVYPSO algorithm.

Through PSO-guided global movement and ivy-inspired local perturbations, IVYPSO forms a complementary hybrid search system. The use of ivy growth variables enhances the adaptability and solution refinement, particularly in rugged or complex landscapes. The implementation process of IVYPSO is illustrated in Figure 4 and Algorithm 1.
**Algorithm 1.** IVYPSOInput: N, Max_iteration, lb, ub, dim, fobjOutput: Destination_fitness, Destination_position, Convergence_curveInitialize parameters:    Set PSO parameters: inertia weight (w), cognitive factor (c1), social factor (c2)    Initialize population size (N), maximum iterations (Max_iteration), search space (lb, ub)    Define and initialize the vine growth variable (GV)Initialize population:    For each particle i in population:        Randomly initialize position Position_i within [lb, ub]        Initialize velocity Velocity_i as a zero vector        Evaluate fitness Cost_i = fobj(Position_i)        Initialize vine growth variable GV_i = Position_i/(ub - lb)        Set personal best PBest_i = Position_i and PBest_Cost_i = Cost_i    Set global best GBest as the particle with the lowest fitness valueIteration loop (t = 1 to Max_iteration):    For each particle i in population:        Update velocity and position:            Generate random vectors r1 and r2            Velocity_i = w * Velocity_i                 + c1 * r1 * (PBest_i - Position_i)                 + c2 * r2 * (GBest - Position_i)        Calculate dynamic control factor β:          β = 1 + (random/2)        Perform local or global search based on fitness comparison:          If Cost_i < β * GBest_Cost:            New_Position = Position_i                     + |N(0,1)| * (Position_neighbor - Position_i)                     + N(0,1) * GV_i          Else:            New_Position = GBest * (random + N(0,1) * GV_i)        Boundary handling:          Ensure New_Position is within [lb, ub]        Update vine growth variable:          GV_i = GV_i * (random^2 * N(0,1))        Evaluate and update solutions:          New_Cost = fobj(New_Position)          If New_Cost < Cost_i:            Position_i = New_Position            Cost_i = New_Cost            If New_Cost < PBest_Cost_i:                PBest_i = New_Position                PBest_Cost_i = New_Cost                If New_Cost < GBest_Cost:                    GBest = New_Position                    GBest_Cost = New_Cost    Record the best fitness at the current iteration:        Convergence_curve(t) = GBest_CostReturn:    Destination_fitness = GBest_Cost    Destination_position = GBest    Convergence_curve

## 5. Results and Analytical Evaluation of the Experiment

To verify the proposed algorithm’s reliability and performance, 26 standard benchmark functions were utilized. Moreover, we applied the algorithm to three real-world engineering optimization problems to evaluate its practical performance. The subsequent sections offer comprehensive details on the benchmark functions, parameter configurations, and performance metrics. The algorithm’s effectiveness was assessed through a comparative analysis with ten widely recognized metaheuristic algorithms.

Setup for experiments: The proposed IVYPSO algorithm and other metaheuristic methods were implemented in MATLAB 2023a. All tests were conducted on a Windows 10 platform with an Intel(R) Core (TM) i9-14900KF processor (3.10 GHz) and 32 GB of RAM.

### 5.1. Global Optimization with 26 Benchmark Mathematical Test Functions

To assess the performance of the proposed IVYPSO algorithm in solving complex optimization problems, this study employed 26 widely recognized benchmark functions [34,35,36]. These functions were selected to ensure a comprehensive evaluation covering various optimization scenarios, and they are grouped here into two main categories: unimodal and multimodal functions. Each function was tested in a 30-dimensional space.

**Unimodal functions:** The initial set of 15 benchmark functions (F1–F15) is unimodal, characterized by the presence of a single global optimum, making it suitable for evaluating the convergence speed and local exploitation capability of optimization algorithms. These functions provide a smooth search landscape without local optima, allowing the assessment of an algorithm’s ability to quickly converge to the global minimum.

For instance, the Quartic function introduces a noise component that simulates real-world measurement errors, making it relevant for applications such as experimental data fitting. The Sum Power function, with its amplified penalization of higher-order dimensions, reflects challenges seen in structural reliability or robust design tasks in engineering optimization.

**Multimodal functions:** The latter set of functions (F16–F26) is multimodal, containing numerous local optima and being employed to test an algorithm’s ability to maintain diversity and avoid premature convergence. These functions simulate complex landscapes typically encountered in real-world scenarios such as material design, resource allocation, or non-linear process optimization.

For example, the Alpine function represents rugged fitness landscapes with a repetitive pattern, akin to multi-peak phenomena in signal processing or energy system optimization. The Weierstrass function, characterized by its fractal-like structure, is often used to test algorithms under highly irregular and non-differentiable conditions, making it applicable to domains such as financial modeling or dynamic system tuning.

By adopting this comprehensive set of 26 benchmark functions, this study evaluated IVYPSO across a wide range of conditions, from simple landscapes to highly complex multimodal terrains. This thorough testing ensures that the algorithm’s effectiveness, robustness, and generalization ability were rigorously validated. The full mathematical formulations and parameter settings of the benchmark functions are detailed in Table 1.

#### 5.1.1. Performance Indicators

To objectively assess the effectiveness of the IVYPSO algorithm, this study used the following standard evaluation metrics to comprehensively assess its performance across different benchmark tests [37,38,39,40].

Average value (Avg): The average fitness value obtained from M independent runs of the algorithm, calculated as shown in Equation (21).

(21) $$Avg=\frac{\sum_{i=1}^{M}\;(f_{i})}{M}$$

Standard deviation (Std): The variability in the objective function values obtained from M independent runs of the algorithm. The standard deviation is calculated using Equation (22).

(22) $$Std=\sqrt{\frac{1}{M-1}\sum_{i=1}^{M}\;(f_{i}-Avg{)}^{2}}$$

Best: The minimum fitness obtained from M independent runs of the algorithm, as shown in Equation (23).

(23) $$Best=\underset{1\leq i\leq M}{min}\;f_{i}$$

where $f_{i}$ denotes the optimal fitness value attained during run i.

#### 5.1.2. Parameter Settings and Performance Comparison Against Other Algorithms

Table 2 summarizes the parameter settings for the IVYPSO algorithm and its comparison algorithms. The proper adjustment of the parameters for each algorithm is crucial to ensure optimal performance. To maintain the fairness of the comparison, the initial settings and common parameters for all algorithms are set based on the standard values from the existing literature, while the remaining parameters are optimized through experimental procedures. The comparison algorithms selected in this study include classic metaheuristic methods such as PSO, IVY, BOA [41], and WOA [42], as well as several recently proposed improved algorithms, including GOOSE and HPSOBOA, and several advanced variant algorithms such as FDC-AGDE, dFDB-LSHADE, NSM-BO, dFDB-SFS, and FDB-AGSK. These algorithms have demonstrated strong performance in the IEEE CEC competition and in solving real-world engineering optimization problems. In this study, the evaluation of the objective function is terminated upon reaching the maximum number of iterations.

#### 5.1.3. Analysis of Numerical Results

The proposed algorithm’s performance was evaluated and compared with several mature and latest algorithms.

Table 3 presents a detailed comparison of the average fitness values and standard deviations achieved by the IVYPSO algorithm and other algorithms across the test functions. Notably, IVYPSO attained the best average fitness on 21 out of the 26 test functions (F1–F4, F6, F8–F20, F24–F26), outperforming the other 11 algorithms. Particularly impressive was its performance on 17 functions (F1–F4, F8, F9, F11, F13–F17, F19, F20, F24–F26), where it achieved the best average fitness with a standard deviation of zero, demonstrating excellent stability and efficiency. On functions F5, F21, and F23, the IVYPSO algorithm ranked 6th, 5th, and 4th, respectively. However, its performance on F7 and F22 was relatively poor, ranking 11th and 12th, respectively.

Table 4 compares the best fitness values obtained by the IVYPSO algorithm and the other 11 algorithms across the test functions. Among the 26 functions, IVYPSO achieved superior results on 20 functions (F1–F4, F8, F9, F11–F20, F24–F26), outperforming the other 11 algorithms. For functions F6, F21, and F23, IVYPSO ranked 2nd, 4th, and 2nd, respectively, in terms of the best fitness value. Nevertheless, its performance was inferior on functions F5, F7, and F22, where it ranked 8th, 11th, and 10th, respectively.

In summary, the IVYPSO algorithm can be considered a superior optimization algorithm.

#### 5.1.4. Analysis of Convergence Behavior

**Figure 5:**
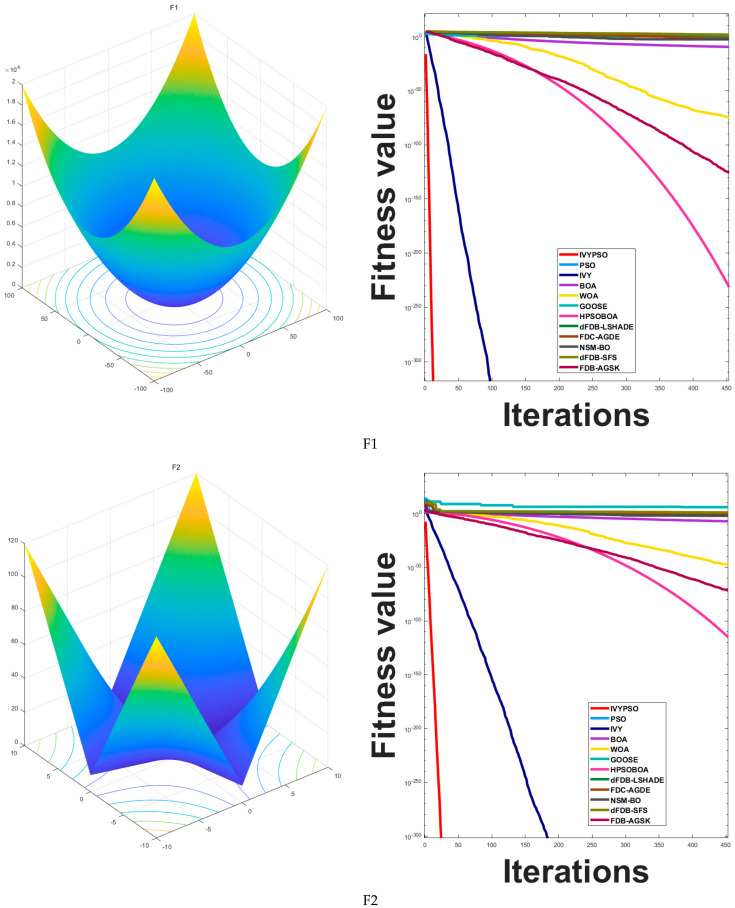

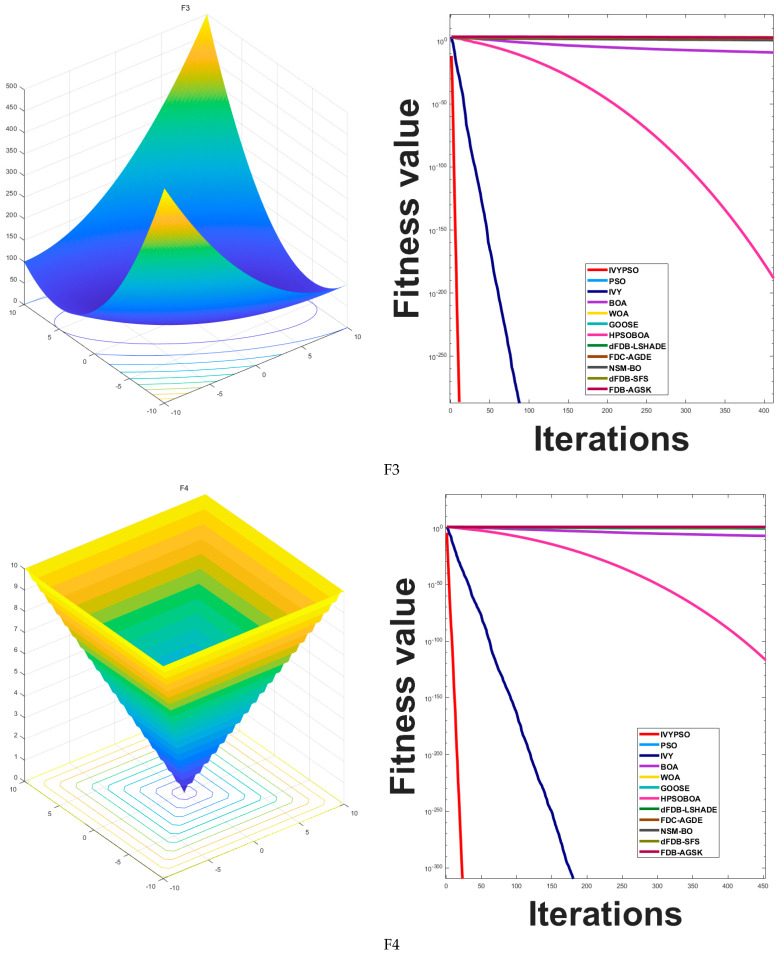

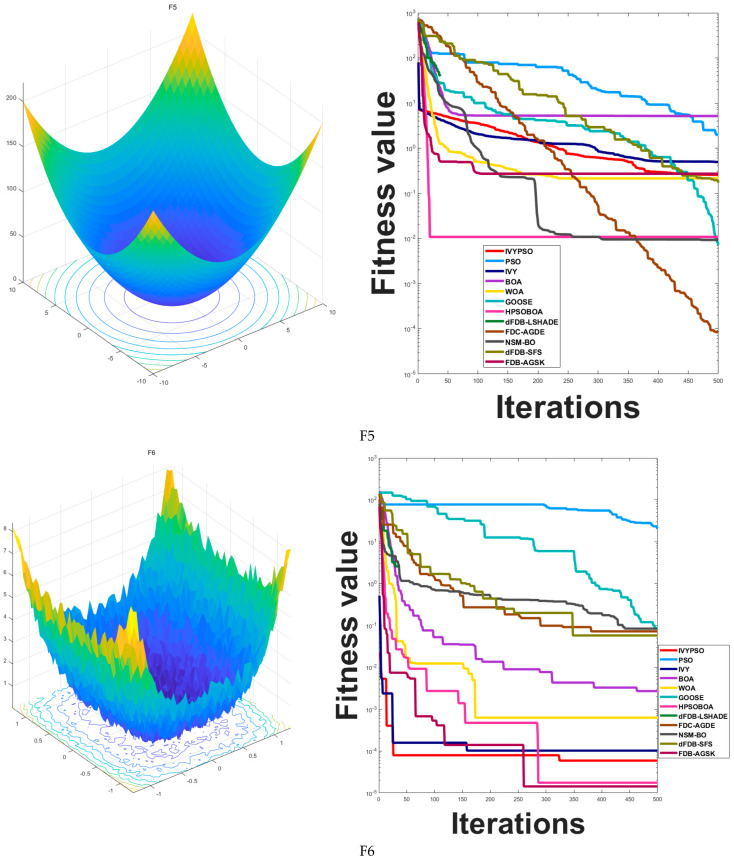

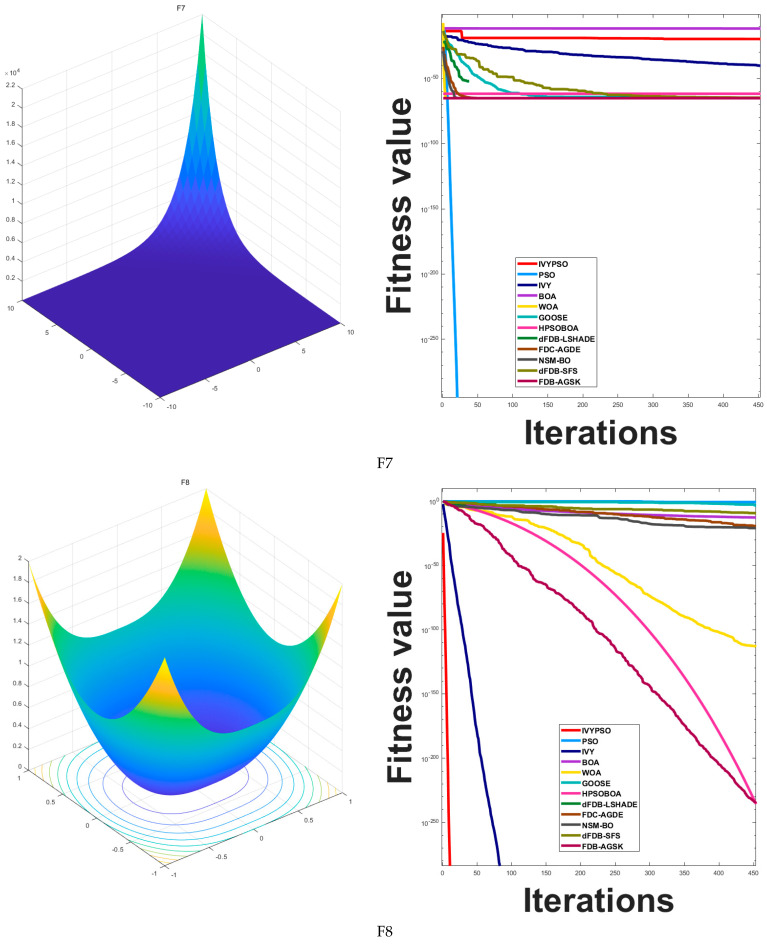

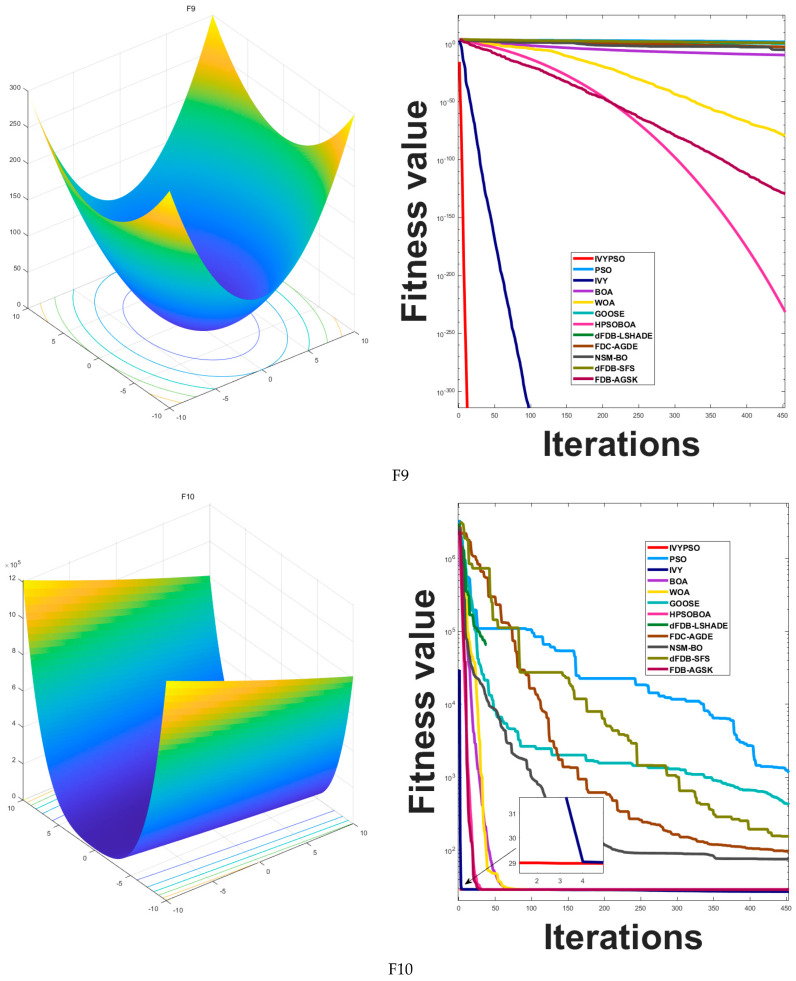

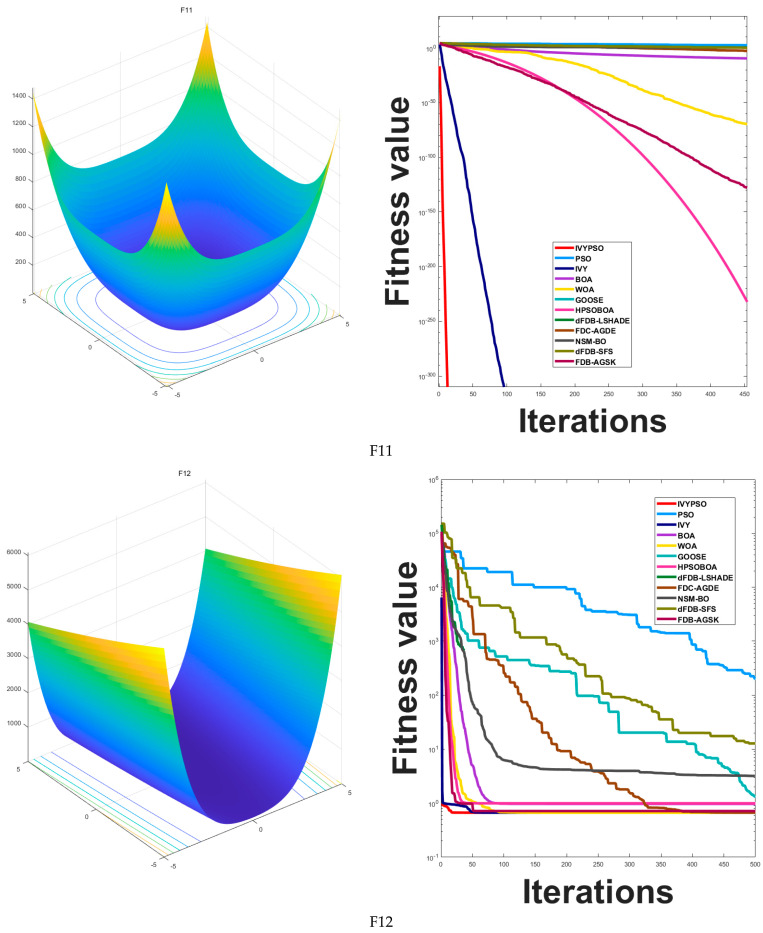

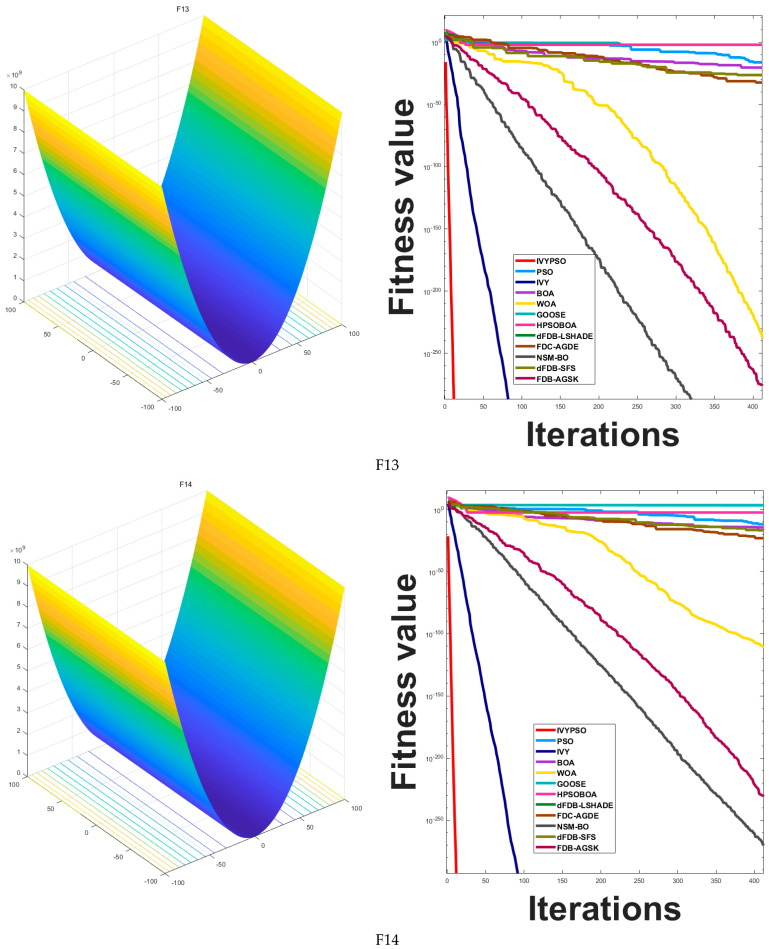

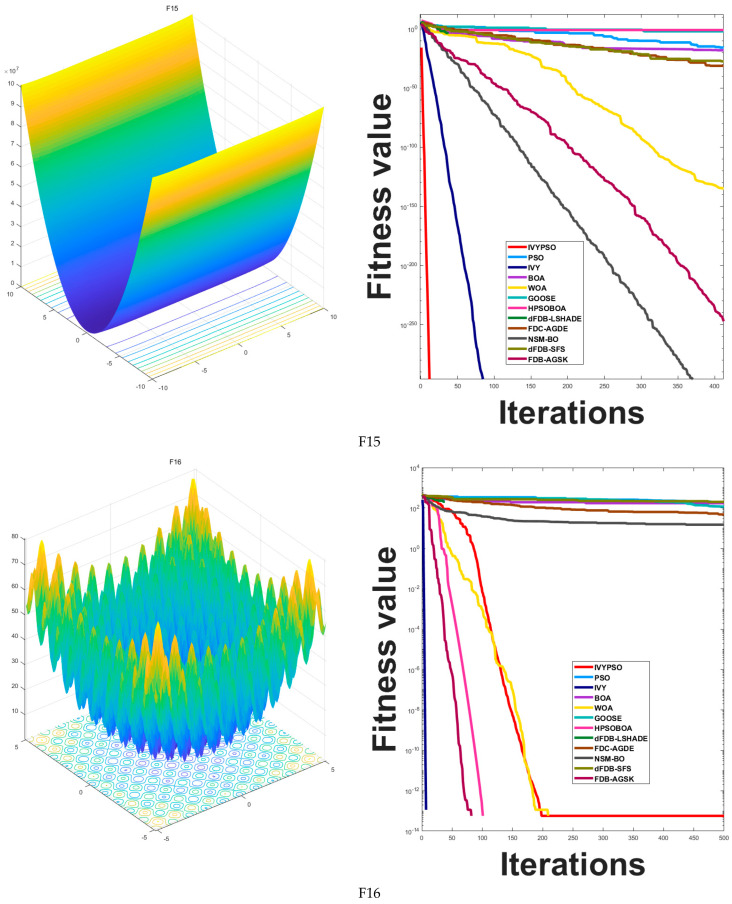

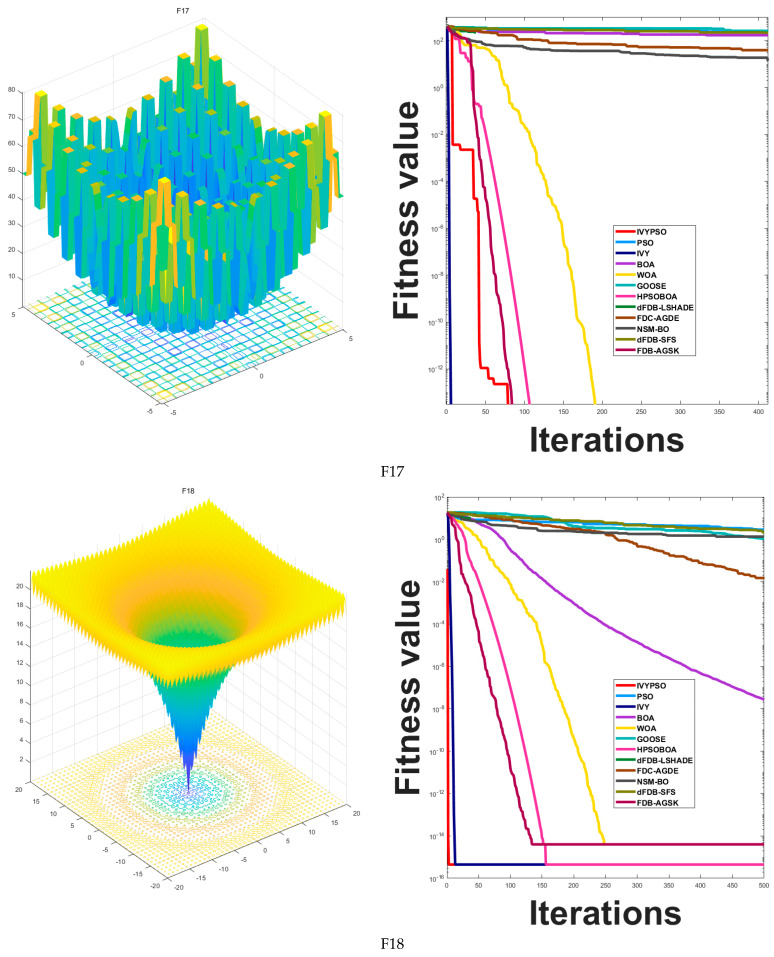

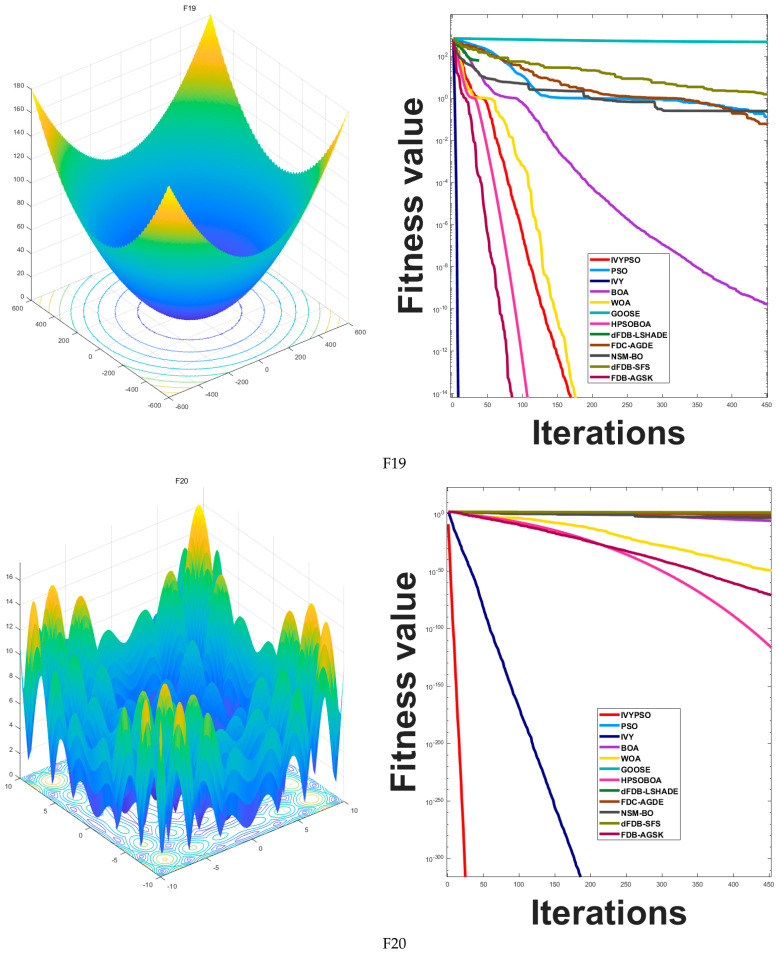

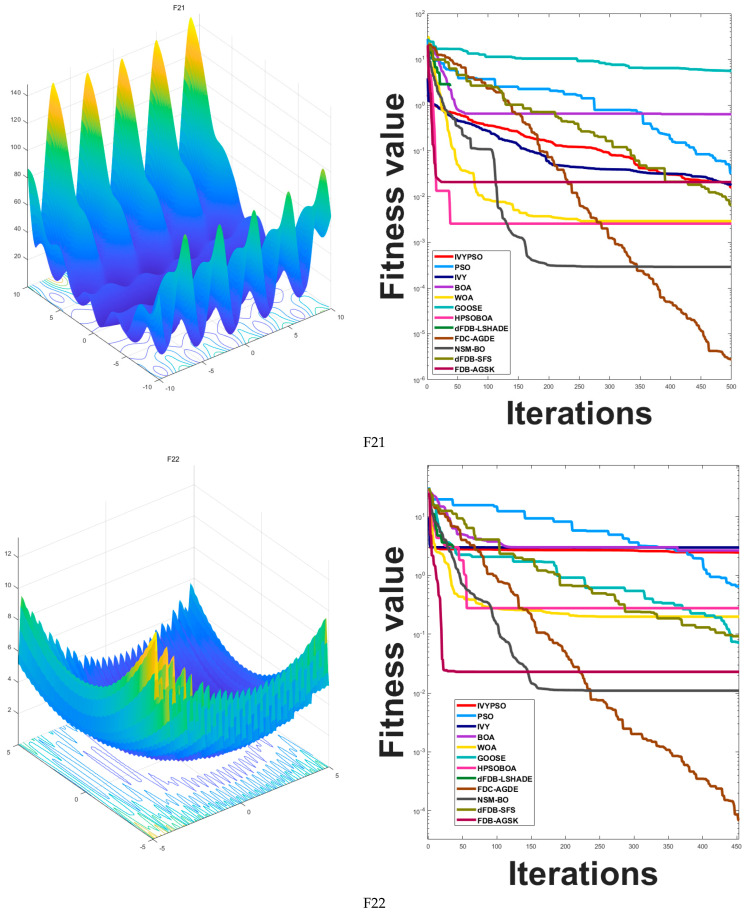

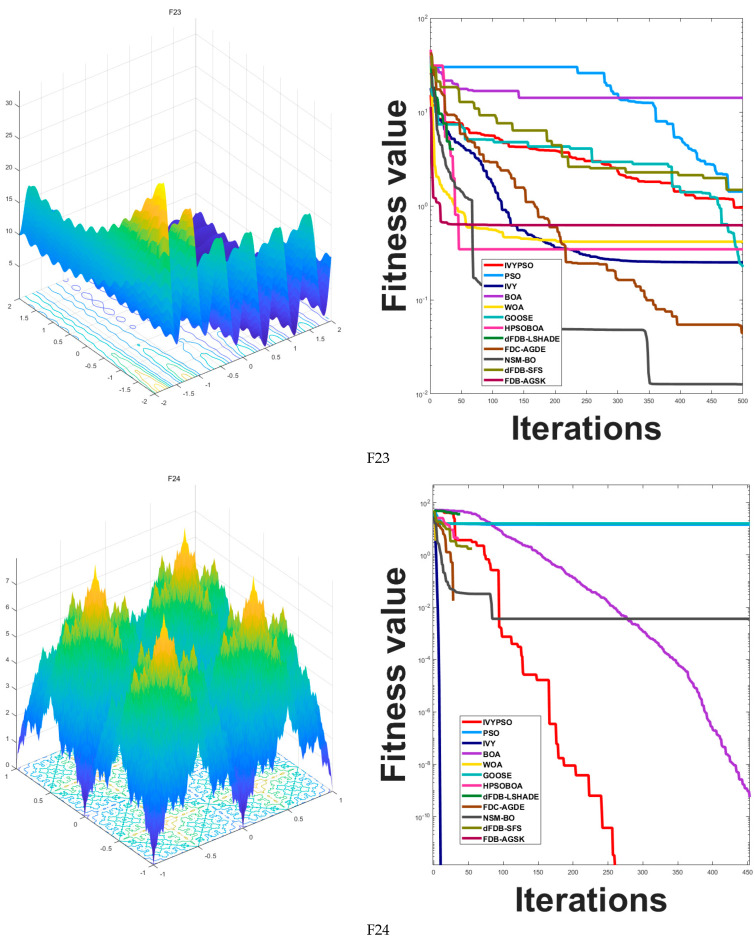

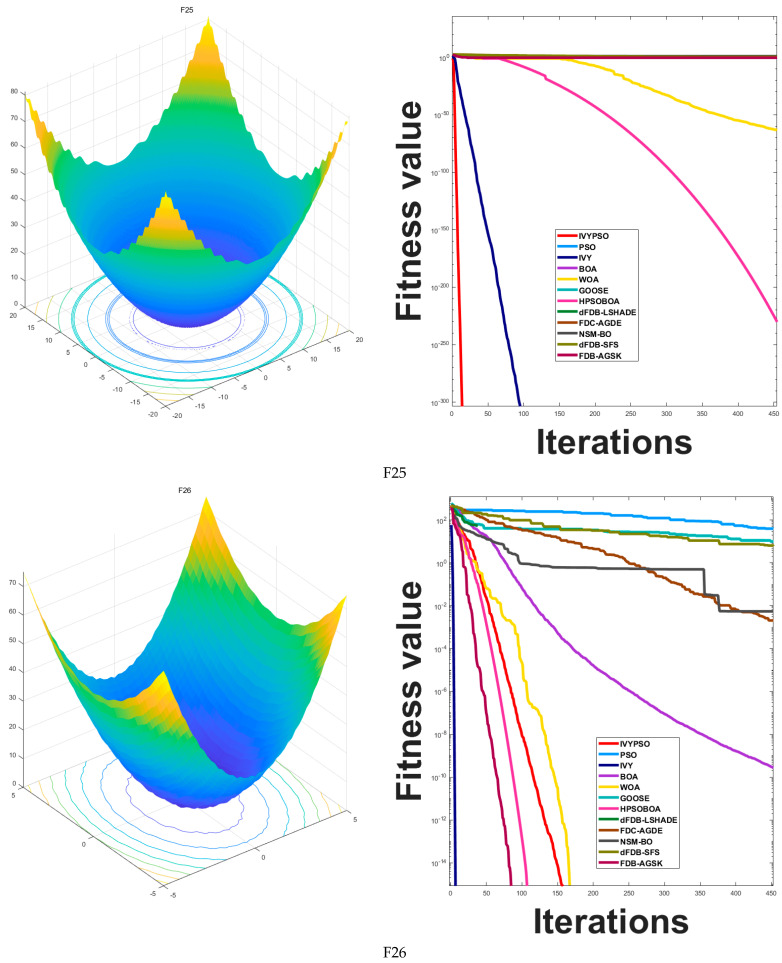
The convergence behaviors of IVYPSO and other algorithms for various test functions.

Figure 5 compares the convergence behavior of the IVYPSO algorithm with that of other algorithms over 500 iterations. The vertical axis represents the best fitness value obtained at each iteration, while the horizontal axis indicates the iteration count. On 16 out of the 26 test functions (specifically F1–F4, F6, F8–F15, F18, F20, and F25), IVYPSO demonstrated consistently faster and more efficient convergence, outperforming the other algorithms. Although IVYPSO exhibits strong global optimization capability, a few other algorithms outperformed it in certain specific cases.

#### 5.1.5. Analysis of Exploitation Capabilities

Ideal for evaluating the algorithm’s development capabilities, the single-modal benchmark functions (F1–F14) contain only one minimum. For functions F1–F4, F8, F9, F11, F13, and F14, Figure 3 demonstrates that IVYPSO reaches the theoretical best solution in around 50 iterations. Reflecting IVYPSO’s high precision and stability, Table 3 reveals that the average fitness and standard deviation for these functions are typically 0. These findings clearly indicate that IVYPSO outperforms most of the comparison algorithms on the benchmark functions. For the multi-modal functions F15–F17, F19, F20, and F24–F26, IVYPSO also attains the theoretical best fitness in around 50 iterations. Table 4 demonstrates that the majority of the algorithms are capable of locating the theoretical optimal solution. Moreover, Table 3 underscores that IVYPSO demonstrates better average fitness and standard deviation than most competing algorithms, highlighting its robust and stable exploration capabilities.

#### 5.1.6. Wilcoxon Signed-Rank Analysis Results and Friedman Ranking Scores

To ensure statistically robust conclusions, the widely accepted Wilcoxon non-parametric [43,44,45,46] test was used to assess the effectiveness of IVYPSO compared to 11 other algorithms. Table 5 presents the results of the Wilcoxon signed-rank test applied to 26 standard test functions at a significance level of α = 0.05, using the mean objective value of each function as the test sample. The *p*-value, which reflects the significance level, is considered significant when below 0.05. The data in Table 5 show that IVYPSO yields *p*-values below 0.05 in all cases. Therefore, this analysis statistically validates the superiority of IVYPSO over the other 11 algorithms.

To evaluate further the overall performance of the proposed IVYPSO algorithm on the 26 benchmark functions, the Friedman scores were used to rank the average performances of 12 comparative algorithms [47,48,49]. Table 6 presents the Friedman scores and their corresponding ranks. Among all algorithms, IVYPSO achieved the lowest Friedman score of 1.9231, ranking first, which demonstrates its outstanding performance across all test functions. In contrast, traditional algorithms such as PSO and BOA obtained Friedman scores of 5.8846 and 6.7308, ranking 9th and 11th, respectively, indicating relatively weaker overall performance. Similarly, some improved algorithms, such as FDC-AGDE and dFDB-LSHADE, ranked 12th and 10th, respectively, also underperforming compared to IVYPSO. These results indicate that IVYPSO exhibits strong robustness and competitiveness in solving complex optimization problems, and its overall performance surpasses that of both traditional and newly developed metaheuristic algorithms.

#### 5.1.7. Analysis of Computational Expenses

The time complexity of the IVYPSO algorithm is primarily composed of three parts: the population initialization, iteration updates, and fitness evaluation. Let the population size be N, the maximum number of iterations be T, and the dimensionality of the decision variables be dim. In the population initialization phase, the random generation of each particle’s position and velocity, along with the fitness evaluation, requires a time complexity of O(N×dim). In each iteration, the algorithm updates the velocity and position of each particle, adjusts the ivy growth variables, and evaluates the fitness. The velocity and position updates involve basic vector operations, with a time complexity of O(dim), and the ivy growth variable update and random perturbation for local or global searches also have a time complexity of O(dim). The fitness evaluation is usually the most time-consuming operation. If the complexity of the objective function evaluation is O(f), the fitness calculation for the entire population in each iteration has a time complexity of O(N×f). Therefore, the overall time complexity of the IVYPSO algorithm can be expressed as O(T×N×(dim+f)). As the population size N, maximum iteration count T, and dimensionality dim increase, the algorithm’s time complexity grows linearly, making it suitable for large-scale, complex global optimization problems.

### 5.2. Application of IVYPSO to Engineering Optimization Problems

This section presents the application of the IVYPSO algorithm in solving engineering optimization problems that involve various inequality and equality constraints. For each optimization problem, IVYPSO was evaluated in 20 independent runs using a population size of 30 individuals, with a maximum of 500 iterations. The performance of IVYPSO was compared with 11 other algorithms, including PSO, IVY, BOA, WOA, GOOSE, HPSOBOA, FDC_AGDE, dFDB_LSHADE, NSM_BO, dFDB_SFS, and FDB_AGSK. Additionally, three stability analysis metrics were incorporated: SR, ACTs, and AFEs. SR represents the proportion of independent runs in which the algorithm successfully found solutions that satisfy all constraints and reach the global optimum or an acceptable level of precision. ACTs refers to the average time (in seconds) spent by the algorithm to complete each optimization task across multiple runs. AFEs indicates the average number of fitness function evaluations required to complete the optimization task over multiple experiments.

#### 5.2.1. Gas Transmission Compressor Design (GTCD) Problem

**Figure 6:**
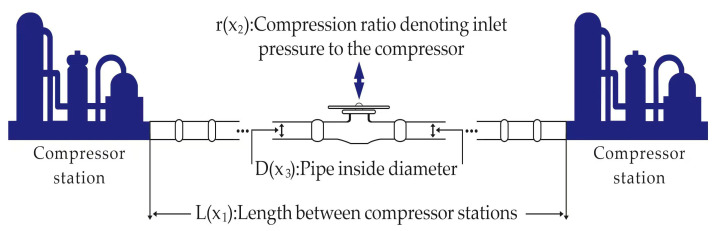
A gas pipeline transmission system for the GTCD problem.

Figure 6 illustrates the gas transmission compressor design (GTCD) problem, which is a representative and practical mechanical design case originally proposed by Beightler and Phillips [50]. This problem involves determining the optimal values of several design variables, such as the pipeline length, inlet and outlet pressures, and pipe diameter, with the objective of minimizing the total cost of a gas pipeline transmission system while ensuring the delivery of 100 million cubic feet of natural gas per day.

The GTCD problem reflects a realistic and complex engineering scenario widely encountered in energy and process industries. It poses significant optimization challenges due to its non-linear, constrained, and multimodal nature. By applying the proposed IVYPSO algorithm to this problem, we aim to demonstrate its capability to handle real-world design constraints, achieve cost-effective solutions, and maintain robustness in practical optimization tasks. This problem has three decision variables: the length L between two compressor stations; the compression ratio L, at the compressor inlet, where $r=\frac{P_{1}}{P_{2}}$, with $P_{1}$ being the pressure leaving the compressor station (psi) and $P_{2}$ being the pressure entering the compressor station (psi); and D, the internal diameter of the pipeline (inches). The goal is to find the optimal values for L, r, and D that minimize the $C_{1}$ value.

In this problem, the total annual cost of the gas transmission system is defined as in Equation (24):

(24) $$\begin{array}{cc} minC_{1}(x)= & 8.61\times 10^{5}{x_{3}}^{-\frac{2}{3}}{x_{1}}^{\frac{1}{2}}x_{2}{\left({x_{2}}^{2}-1\right)}^{-\frac{1}{2}}\\ & +3.69\times 10^{4}x_{3}-7.6543\times 10^{8}{x_{1}}^{-1}\\ & +7.72\times 10^{8}{x_{1}}^{-1}{x_{2}}^{0.219}, \end{array}$$

where $x_{1}=L$, $x_{2}=r$, $x_{3}=D$, subject to:

$$10\leq x_{1}\leq 55,$$

$$1.1\leq x_{2}\leq 2,$$

$$10\leq x_{3}\leq 40.$$

Table 7 presents a comparison of the results for solving the gas transmission compressor design problem using IVYPSO and other algorithms from the literature. The success rate threshold for this problem was determined to be 1,677,759.2755. The best result achieved by IVYPSO was L=24.4960, r=1.5867, D=20.0000, with a minimum cost of 1,677,759.2755 and with an SR value of 100%, which required minimal time to complete the task. Table 8 shows a statistical analysis, showing that IVYPSO achieved the lowest average cost of 1,677,759.2755. These results demonstrate that compared to other optimization algorithms, IVYPSO delivers a superior solution to the gas transmission compressor design problem.

#### 5.2.2. Three-Bar Truss Design Problem

**Figure 7:**
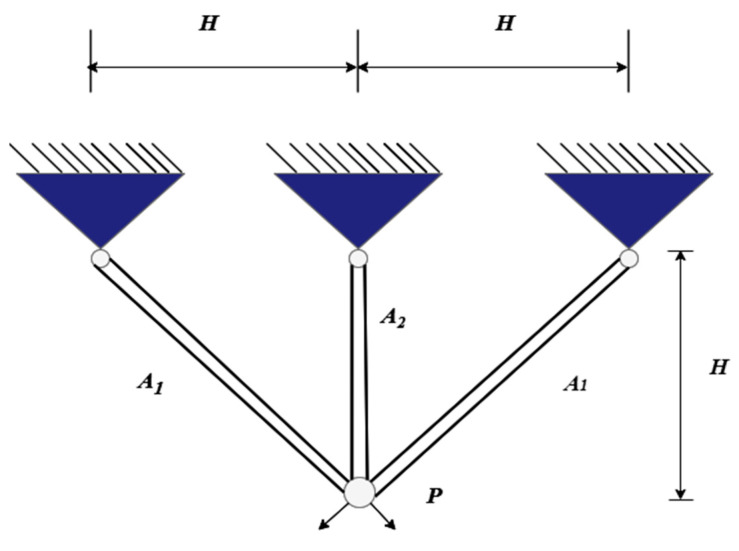
Schematic diagram of the three-bar truss design.

Figure 7 shows the three-bar truss design problem. The three-bar truss is typically a simple planar truss structure composed of three rods forming a triangular shape. This problem is widely applicable in engineering design, particularly in the field of structural optimization. The design variables typically include the cross-sectional area or dimensions of the rods, with the objective of minimizing the total mass of the truss while ensuring compliance with stress and geometric constraints. Such problems effectively reflect real-world engineering demands for structural safety and material efficiency, making them commonly used as benchmark problems for evaluating the performance of optimization algorithms.

The total mass of the three-bar truss can be expressed by Equation (25):

(25) $$minf(x)=\left(2\sqrt{2}x_{1}+x_{2}\right)\times H$$

where $x_{1}=A_{1}$, $x_{2}=A_{2}$:

$$g_{1}(x)=\frac{\sqrt{2}x_{1}+x_{2}}{\sqrt{2}x_{1}+\frac{2x_{1}x_{2}}{x_{2}}}P-\sigma \leq 0,$$

$$g_{2}(x)=\frac{P}{\sqrt{2}x_{1}+\frac{2x_{1}x_{2}}{x_{2}}}-\sigma \leq 0,$$

$$g_{3}(x)=\frac{P}{x_{1}+\sqrt{2}x_{2}}-\sigma \leq 0,$$

$$0\leq x_{1},x_{2}\leq 1.$$

where $H=1000mm,P=2{kN/cm}^{2},\sigma =2{kN/cm}^{2}$.

Table 9 presents a comparison of the results for solving the three-bar truss design problem using IVYPSO and other algorithms from the literature. The success rate threshold for this problem was determined to be 263.8523. The best result obtained by IVYPSO was $A_{1}=0.7884$, $A_{2}=0.4081$, with a minimum cost of 263.8523 and an SR value of 100%, and it required minimal time to complete the task. Table 10 provides a statistical analysis, showing that IVYPSO achieved the lowest average cost of 263.8523. These results indicate that compared to other optimization algorithms, IVYPSO provides a better solution for the three-bar truss design problem.

#### 5.2.3. Multiple-Disk Clutch Brake Design Problem

**Figure 8:**
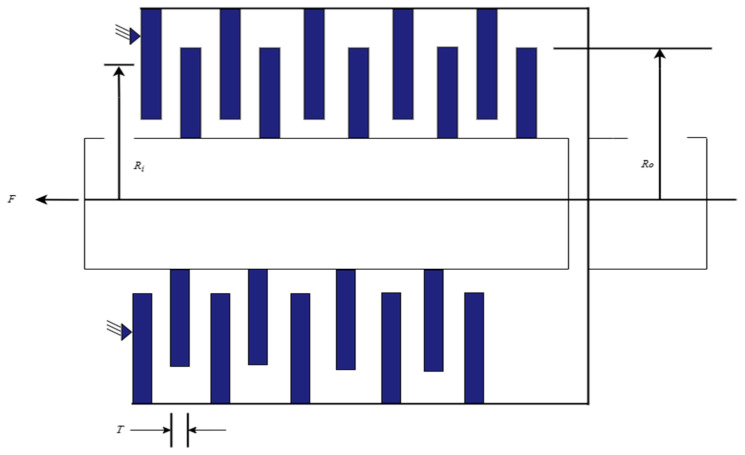
Schematic view of multiple-disk clutch brake design problem.

Figure 8 illustrates the multiple-disk clutch brake design problem, a classic engineering optimization issue that is commonly encountered in automation equipment, mechanical transmission systems, and the automotive industry [51]. This problem involves optimizing the design of the clutch and brake system to minimize the stopping time of the brake while ensuring high operational efficiency and stability. Such design problems are highly relevant to real-world engineering scenarios, where the balance between performance, efficiency, and reliability is critical. By addressing this problem, the algorithm’s ability to handle practical engineering challenges and improve the overall system design is demonstrated, reflecting its applicability in real-world applications. This problem involves five decision variables: the inner radius $r_{i}$ in millimeters, outer radius $r_{o}$ in millimeters, disk thickness t in millimeters, driving force F, and number of friction surfaces Z.

The brake’s stopping time can be expressed by Equation (26):

(26) $$minf(x)=\pi (x_{2}^{2}-x_{1}^{2})x_{3}(x_{5}+1)p_{m}$$

where $x_{1}=r_{i}$, $x_{2}=r_{o}$, $x_{3}=t$, $x_{4}=F$, $x_{5}=Z$, subject to:

$$g_{1}(x)=x_{2}-x_{1}-\Delta R\geq 0$$

$$g_{2}(x)=L_{max}-(Z+1)(t+\delta )\geq 0$$

$$g_{3}(x)=p_{max}-p_{rz}\geq 0$$

$$g_{4}(x)=p_{max}V_{sr,max}-p_{rz}V_{sr}\geq 0$$

$$g_{5}(x)=V_{sr,max}-V_{sr}\geq 0$$

$$g_{6}(x)=M_{h}-sM_{s}\geq 0$$

$$g_{7}(x)=T\geq 0$$

$$g_{8}(x)=T_{max}-T\geq 0$$

$$60\leq x_{1}\leq 80\;mm,90\leq x_{2}\leq 110\;mm,1.5\leq x_{3}\leq 3\;mm,0\leq x_{4}\leq 1000\;N,2\leq x_{5}\leq 9$$

where:

$$p_{m}=0.0000078\;{kg/mm}^{3},p_{max}=1\;MPa,\mu =0.5,V_{sr,max}=10\;m/s,s=1.5,T_{max}=15\;s,$$

$$n=250\;rpm,M_{f}=3\;Nm,I_{z}=55{kg/m}^{2},\delta =0.5\;mm,\Delta R=20\;mm,L_{max}=30\;mm,$$

$$M_{h}=\frac{2}{3}\mu x_{4}x_{5}\frac{x_{2}^{3}-x_{1}^{3}}{x_{2}^{2}-x_{1}^{2}}N\;mm,w=\frac{\pi n}{30}\frac{rad}{s},R_{sr}=\frac{2}{3}\frac{x_{2}^{3}-x_{1}^{3}}{x_{2}^{2}-x_{1}^{2}}mm,A=\pi (x_{2}^{2}-x_{1}^{2}){mm}^{2},$$

$$M_{s}=40\;Nm,p_{rz}=\frac{x_{4}}{A}{N/mm}^{2},V_{sr}=\frac{\pi R_{sr}n}{30}mm/s$$

Table 11 presents a comparison of the results for solving the multiple-disk clutch brake design problem using IVYPSO and other algorithms from the literature. The success rate threshold for this problem was determined to be 0.2352. The best result obtained by IVYPSO was $r_{i}=70$, $r_{o}=90$, t=1, F=1000, Z=2, with the lowest cost of 0.2352 and an SR value of 100%, and it required minimal time to complete the task. Table 12 shows a statistical analysis, showing that IVYPSO achieves the lowest average cost of 0.2352. These results indicate that compared to other optimization algorithms, IVYPSO provides a better solution for the multiple-disk clutch brake design problem.

## 6. Conclusions

The proposed IVYPSO algorithm effectively enhances both the global exploration and local exploitation capabilities within complex search spaces by integrating ivy growth variables and dynamic control factors into the classical PSO framework. The ivy growth variables facilitate diverse search behaviors that prevent premature convergence, while the dynamic control factors adaptively balance the emphasis between global and local searches based on fitness evaluations.

Extensive experiments on 26 benchmark functions and three challenging engineering optimization problems—including the multiple-disk clutch brake design and gas transmission compressor design—demonstrated that IVYPSO achieves superior solution quality with rapid convergence. Comparative analyses against ten state-of-the-art optimization algorithms further confirmed the algorithm’s superiority in convergence accuracy, stability, and robustness, particularly in handling multi-modal and high-dimensional problems where the global optimization ability is critical.

This study lays a foundation for further research in several directions. Our future work will focus on extending IVYPSO to more complex engineering problems and multi-objective optimization scenarios, especially those involving dynamic, multi-modal, and high-dimensional environments. Moreover, the incorporation of adaptive parameter tuning strategies is planned to optimize the design of ivy growth variables and dynamic control factors, thereby improving the algorithm’s flexibility and performance across diverse problem domains.

In addition, hybridizing IVYPSO with other intelligent optimization techniques, such as genetic algorithms, differential evolution, and simulated annealing, to build multi-fusion frameworks may offer significant performance enhancements. This would leverage complementary algorithmic strengths and further improve the solution quality and convergence behavior.

## Figures and Tables

**Table 1:** Details of the 26 test functions.

**s/n**	**Function Name**	Formula	Category	Range	$f_{min}^{*}$
F1	Sphere	$f_{1}(x)=\sum_{i=1}^{dim}\;x_{i}^{2}$	Unimodal	[−100, 100]	0
F2	Schwefel 2.22	$f_{2}(x)=\sum_{i=1}^{dim}\;|x_{i}|+\prod_{i=1}^{dim}\;|x_{i}|$	Unimodal	[−10, 10]	0
F3	Schwefel 1.2	$f_{3}(x)=\sum_{i=1}^{dim}\;{\left(\sum_{j=1}^{i}\;x_{j}\right)}^{2}$	Unimodal	[−100, 100]	0
F4	Schwefel 2.21	$f_{4}(x)=\underset{i}{max}\;\{|x_{i}|\},1\leq i\leq dim$	Unimodal	[−100, 100]	0
F5	Step	$f_{5}(x)=\sum_{i=1}^{dim}\;{\left(x_{i}+0.5\right)}^{2}$	Unimodal	[−100, 100]	0
F6	Quartic	$f_{6}(x)=\sum_{i=1}^{dim}\;ix_{i}^{4}+rand$	Unimodal	[−1.28, 1.28]	0
F7	Exponential	$f_{7}(x)=\sum_{i=1}^{dim}\;(e^{x_{i}}-x_{i})$	Unimodal	[−10, 10]	0
F8	Sum power	$f_{8}(x)=\sum_{i=1}^{dim}\;x_{i}^{2}$	Unimodal	[−1, 1]	0
F9	Sum square	$f_{9}(x)=\sum_{i=1}^{dim}\;ix_{i}^{2}$	Unimodal	[−10, 10]	0
F10	Rosenbrock	$f_{10}(x)=\sum_{i=1}^{dim-1}\;\left(100(x_{i+1}-x_{i}^{2}{)}^{2}+(x_{i}-1{)}^{2}\right)$	Unimodal	[−5, 10]	0
F11	Zakharov	$f_{11}(x)=\sum_{i=1}^{dim}\;x_{i}^{2}+{\left(\sum_{i=1}^{dim}\;0.5ix_{i}\right)}^{2}+{\left(\sum_{i=1}^{dim}\;0.5ix_{i}\right)}^{4}$	Unimodal	[−5, 10]	0
F12	Trid	$f_{12}(x)=\sum_{i=1}^{dim}\;(x_{i}-1{)}^{2}-\sum_{i=2}^{dim}\;x_{i}x_{i-1}$	Unimodal	[−5, 10]	0
F13	Elliptic	$f_{13}(x)=\sum_{i=1}^{dim}\;(10^{6}{)}^{i/(dim-1)}x_{i}^{2}$	Unimodal	[−100, 100]	0
F14	Cigar	$f_{14}(x)=x_{1}^{2}+10^{6}\sum_{i=2}^{dim}\;x_{i}^{2}$	Unimodal	[−100, 100]	0
F15	Rastrigin	$f_{15}(x)=\sum_{i=1}^{dim}\;\left(x_{i}^{2}-10cos(2\pi x_{i})+10\right)$	Fixed	[−5.12, 5.12]	0
F16	NCRastrigin	$f_{16}(x)=\sum_{i=1}^{dim}\;\left(x_{i}^{2}-10cos(2\pi x_{i})+10\right),y_{i}=\left\{\begin{array}{c} x_{i},\;\;\;\;\;\;\;\;ifx_{i}\leq 0.5\\ x_{i}-1,\;\;\;\;otherwise\;\;\;\; \end{array}\right.$	Multimodal	[−5.12, 5.12]	0
F17	Ackley	$f_{17}(x)=20e^{-0.2\sqrt{\frac{1}{dim}\sum_{i=1}^{dim}\;x_{i}^{2}}}+e^{-1}\sum_{i=1}^{dim}\;cos(2\pi x_{i})+20+e$	Multimodal	[−50, 50]	0
F18	Griewank	$f_{18}(x)=1+\frac{1}{4000}\sum_{i=1}^{dim}\;x_{i}^{2}-\prod_{i=1}^{dim}\;cos\left(\frac{x_{i}}{\sqrt{i}}\right)$	Multimodal	[−600, 600]	0
F19	Alpine	$f_{19}(x)=\sum_{i=1}^{dim}\;|x_{i}sin(x_{i})+0.1x_{i}|$	Fixed	[−10, 10]	0
F20	Penalized 1	$f_{20}\left(x\right)=\frac{\pi }{dim}\left\{10{sin}^{2}(\pi y_{1})+\sum_{i=1}^{dim-1}\;(y_{i}-1{)}^{2}[1+10{sin}^{2}(\pi y_{i+1})]\right.\left.+(y_{dim}-1{)}^{2}\right\}+\;\sum_{i=1}^{dim}\;u\left(x_{i},10,100,4\right),y_{i}=1+\frac{x_{i}+1}{4},u(x_{i},a,k,m)=\left\{\begin{array}{c} k(x_{i}-a{)}^{m},\;\;\;\;x_{i}>a\\ 0,\;\;\;\;\;\;\;\;\;\;\;\;\;-a\leq x_{i}\leq a\\ k(-x_{i}-a{)}^{m},\;\;\;x_{i}<-a\;\;\;\;\;\;\;\; \end{array}\right.$	Multimodal	[−100, 100]	0
F21	Penalized 2	$f_{21}(x)=0.1\left\{{sin}^{2}(3\pi x_{1})+\sum_{i=1}^{dim-1}\;(x_{i}-1{)}^{2}[1+{sin}^{2}(3\pi x_{i+1})]\right.+(x_{dim}-1{)}^{2}[1\;+{sin}^{2}(2\pi x_{dim})]\}+\sum_{i=1}^{dim}\;u(x_{i},5,100,4)$	Multimodal	[−100, 100]	0
F22	Schwefel	$f_{22}(x)=\sum_{i=1}^{dim}\;x_{i}sin(\sqrt{\left|x_{i}\right|})$	Fixed	[−100, 100]	0
F23	Lévy	$f_{23}(x)={sin}^{2}(3\pi x_{1})+\sum_{i=1}^{dim}\;(x_{i}-1{)}^{2}[1+{sin}^{2}(3\pi x_{i+1})]+(x_{dim}-1{)}^{2}[1+{sin}^{2}(2\pi x_{dim})]$	Multimodal	[−10, 10]	0
F24	Weierstrass	$f_{24}(x)=\sum_{i=1}^{dim}\;\left(\sum_{k=0}^{k_{max}}\;a^{k}cos(2\pi b^{k}(x_{i}+0.5))\right)-dim\left(\sum_{k=0}^{k_{max}}\;a^{k}cos(\pi b^{k})\right),a=0.5,\;b=3,k_{max}=20$	Multimodal	[−0.5, 0.5]	0
F25	Solomon	$f_{25}(x)=1-cos\left(2\pi \sqrt{\sum_{i=1}^{dim}\;x_{i}^{2}}\right)+0.1\sqrt{\sum_{i=1}^{dim}\;x_{i}^{2}}$	Fixed	[−100,100]	0
F26	Bohachevsky	$f_{26}(x)=\sum_{i=1}^{dim}\;\left(x_{i}^{2}+2x_{i}^{2}-0.3cos(3\pi x_{i})\right)$	Fixed	[−10,10]	0

**Table 2:** Parameter settings of IVYPSO and other algorithms.

**Algorithm**	Parameter	Algorithm	Parameter
ALL	Max iteration = 500, Agents = 30, Runs = 30	HPSOBOA	$\begin{array}{c} w=0.7,a=(0.1,0.3),V=(-1,1),C_{1}=\\ C_{2}=0.5,c_{0}=0.01,p=0.6 \end{array}$
IVYPSO	$C_{1}=C_{2}=1.5,w=0.7,beta_{1}=\left[1,\;1.5\right),GV=[0,1]$	dFDB_LSHADE	p_best_rate=0.11, arc_rate=1.4 memory_size=5,memory_pos=1
PSO	$C_{1}=C_{2}=2,V=(-6,6),w=(0.2,0.9)$	FDC_AGDE	$NW=\left[0.5\;0.5\right],Cr\_All=zeros(1,2)$
IVY	$beta_{1}=\left[1,1.5\right),\;GV=[0,1]$	NSM_BO	$p_{{xgm}_{initial}}=0.03,scab=1.25,scsb=1.3$ rcpp=0.0035,tsgs_factor_max=0.05
BOA	$a=0.1,p=0.6,c_{0}=0.01$		
WOA	$\begin{array}{c} a=linear\;decrease\;from\;2\;to\;0,\\ C=[0,2],a2=linear\;decrease\;from-1\;to-2 \end{array}$	dFDB_SFS	$d=X\left(A,:\right)-X\left(B,:\right)$
GOOSE	S_W_min=5,S_W_max=25,coe_min=0.17	FDB_AGSK	l=rand()*2−1,b=1

**Table 3:** A comparison of IVYPSO’s average fitness values and standard deviation with 10 other algorithms for various test functions.

Func	Metrics	IVYPSO	PSO	IVY	BOA	WOA	GOOSE	HPSOBOA	dFDB_LSHADE	FDC_AGDE	NSM_BO	dFDB_SFS	FDB_AGSK
F1	Avg	0	2.5159	5.6148 × 10^−160^	7.5380 × 10^−11^	4.4643 × 10^−74^	28.045	1.6444 × 10^−291^	4954.6436	6.1553 × 10^−3^	0.5044	27.5647	6.0516 × 10^−96^
	Std	0	1.1071	8.5891 × 10^−160^	7.0143 × 10^−12^	2.2966 × 10^−73^	103.4571	0	1398.5169	3.6651 × 10^−3^	1.982	10.3053	3.3035 × 10^−95^
	Rank	1	9	3	6	5	11	2	12	7	8	10	4
F2	Avg	0	4.5489	0	2.3029 × 10^−8^	2.5328 × 10^−50^	1359.2299	1.0782 × 10^−145^	43.1042	1.4138 × 10^−2^	4.2033 × 10^−3^	4.5363	3.9126 × 10^−60^
	Std	0	1.1878	0	6.7460 × 10^−9^	1.3725 × 10^−49^	6860.7534	7.0216 × 10^−146^	9.4746	3.4474 × 10^−3^	8.0123 × 10^−3^	1.1547	2.1335 × 10^−59^
	Rank	1	10	1	6	5	12	3	11	8	7	9	4
F3	Avg	0	82.4665	5.6472 × 10^−85^	5.2628 × 10^−11^	422.432	2.43	3.3155 × 10^−292^	188.7533	8.4739	3.6188	174.9405	419.0353
	Std	0	24.2503	4.3479 × 10^−86^	6.0949 × 10^−12^	156.2203	0.8986	0	78.0896	13.0659	3.9852	48.3456	128.6289
	Rank	1	8	3	4	12	5	2	10	7	6	9	11
F4	Avg	0	1.9179	0	2.6176 × 10^−8^	3.8852	0.2502	5.4053 × 10^−147^	4.4093	1.2657	2.2818	1.1509	5.7175
	Std	0	0.32	0	2.4363 × 10^−9^	2.8659	0.2304	3.3527 × 10^−148^	0.744	0.1467	0.4925	0.2463	3.5226
	Rank	1	8	1	4	10	5	3	11	7	9	6	12
F5	Avg	0.241	2.2492	0.4888	5.3766	9.8934 × 10^−2^	0.0114	0.0327	53.3583	6.2779 × 10^−5^	3.2036 × 10^−3^	0.2562	0.5742
	Std	0.1957	1.1515	0.4037	0.4651	7.3668 × 10^−2^	3.7831 × 10^−3^	2.3514 × 10^−2^	16.69	3.1763 × 10^−5^	7.9615 × 10^−3^	8.6912 × 10^−2^	0.5068
	Rank	6	10	8	11	5	3	4	12	1	2	7	9
F6	Avg	6.7137 × 10^−5^	14.4701	6.7440 × 10^−5^	1.9729 × 10^−3^	2.5420 × 10^−3^	0.1336	1.1522 × 10^−4^	1.9443	6.3089 × 10^−2^	0.0984	7.7676 × 10^−2^	1.3547 × 10^−3^
	Std	5.9831 × 10^−5^	10.0623	6.4272 × 10^−5^	8.4663 × 10^−4^	3.0980 × 10^−3^	4.2711 × 10^−2^	8.8115 × 10^−5^	0.8096	1.4809 × 10^−2^	3.9632 × 10^−2^	2.4921 × 10^−2^	2.5808 × 10^−3^
	Rank	1	12	2	5	6	10	3	11	7	9	8	4
F7	Avg	4.8103 × 10^−15^	0	8.7899 × 10^−31^	6.3334 × 10^−11^	7.1751 × 10^−66^	1.2216 × 10^−65^	1.7703 × 10^−62^	1.6598 × 10^−48^	2.5562 × 10^−54^	7.1751 × 10^−66^	2.0229 × 10^−65^	7.1751 × 10^−66^
	Std	1.7831 × 10^−14^	0	4.0250 × 10^−30^	2.0811 × 10^−10^	3.2167 × 10^−81^	1.6721 × 10^−65^	8.7838 × 10^−78^	9.0229 × 10^−48^	1.3999 × 10^−53^	3.2167 × 10^−81^	1.2398 × 10^−65^	3.2167 × 10^−81^
	Rank	11	1	10	12	2	5	7	9	8	3	6	4
F8	Avg	0	0.183	0	9.7665 × 10^−14^	3.5039 × 10^−104^	1.7042 × 10^−5^	1.2449 × 10^−295^	7.6028 × 10^−4^	9.4531 × 10^−20^	2.5527 × 10^−20^	1.4060 × 10^−10^	4.4055 × 10^−153^
	Std	0	0.1684	0	6.2767 × 10^−14^	1.9152 × 10^−103^	1.1127 × 10^−5^	0	1.5541 × 10^−3^	3.4998 × 10^−19^	8.9169 × 10^−20^	3.8320 × 10^−10^	2.3589 × 10^−152^
	Rank	1	12	1	8	5	10	3	11	7	6	9	4
F9	Avg	0	26.7969	4.3686 × 10^−95^	6.8638 × 10^−11^	2.6339 × 10^−73^	0.8848	2.6080 × 10^−291^	658.4361	6.9232 × 10^−4^	0.123	2.9677	1.3465 × 10^−94^
	Std	0	14.5494	0	5.7608 × 10^−12^	1.4319 × 10^−72^	0.7549	0	209.486	4.2068 × 10^−4^	0.4863	0.9921	5.1291 × 10^−94^
	Rank	1	11	3	6	5	9	2	12	7	8	10	4
F10	Avg	26.9025	914.4537	27.6343	28.9104	27.9035	86.349	28.7569	45,111.5779	60.6339	87.2424	162.7128	28.7208
	Std	0.8425	438.8139	0.2558	2.3631 × 10^−2^	0.4628	68.1133	4.3639 × 10^−2^	29,206.2521	43.6974	53.4909	63.2633	9.4422 × 10^−2^
	Rank	1	11	2	6	3	8	5	12	7	9	10	4
F11	Avg	0	111.8508	0	6.6299 × 10^−11^	7.0248 × 10^−75^	0.1491	1.5401 × 10^−291^	679.9458	6.8789 × 10^−4^	9.7422 × 10^−3^	1.7317	2.7436 × 10^−92^
	Std	0	52.0599	0	6.5461 × 10^−12^	2.5674 × 10^−74^	3.6406 × 10^−2^	0	337.3768	3.7684 × 10^−4^	1.8844 × 10^−2^	0.5826	1.5027 × 10^−91^
	Rank	1	11	1	6	5	9	3	12	7	8	10	4
F12	Avg	0.6667	209.9059	0.6667	0.9707	0.667	2.1609	0.9959	1233.9148	0.8234	2.9415	3.9169	0.7818
	Std	9.4147 × 10^−8^	128.9306	4.7975 × 10^−8^	9.8902 × 10^−3^	2.0518 × 10^−4^	1.3914	8.0022 × 10^−4^	870.8841	0.2879	1.8042	1.7217	0.1635
	Rank	1	11	2	6	3	8	7	12	5	9	10	4
F13	Avg	0	1.7597 × 10^−23^	0	1.2000 × 10^−21^	0	3.3757 × 10^−4^	5.2516 × 10^−2^	8.6767	8.4768 × 10^−37^	0	1.8101 × 10^−32^	0
	Std	0	9.2757 × 10^−23^	0	3.9643 × 10^−21^	0	5.2807 × 10^−4^	0.103	17.2354	3.6192 × 10^−36^	0	4.8001 × 10^−32^	0
	Rank	1	8	1	9	1	10	11	12	6	1	7	1
F14	Avg	0	1.2617 × 10^−17^	0	6.5631 × 10^−15^	2.0239 × 10^−103^	1997.0369	0.0359	8.1314	2.6242 × 10^−25^	0	2.3759 × 10^−19^	9.7815 × 10^−151^
	Std	0	4.3869 × 10^−17^	0	3.5821 × 10^−14^	9.0242 × 10^−103^	2107.4933	0.1044	23.3506	1.4323 × 10^−24^	0	5.3197 × 10^−19^	5.3566 × 10^−150^
	Rank	1	8	1	9	5	12	10	11	6	1	7	4
F15	Avg	0	1.0826 × 10^−23^	5.3697 × 10^−260^	1.3855 × 10^−18^	1.1287 × 10^−128^	6.8549 × 10^−3^	3.1054 × 10^−3^	0.0557	1.6634 × 10^−36^	0	1.8751 × 10^−30^	1.6475 × 10^−192^
	Std	0	4.3883 × 10^−23^	6.3684 × 10^−259^	4.4188 × 10^−18^	6.1811 × 10^−128^	1.1247 × 10^−2^	6.5997 × 10^−3^	0.1444	9.0070 × 10^−36^	0	5.4607 × 10^−30^	0
	Rank	1	8	3	9	5	11	10	12	6	1	7	4
F16	Avg	0	169.139	0	26.6377	0	150.3139	0.3609	239.6035	46.6483	10.3208	214.2906	1.8948 × 10^−15^
	Std	0	31.3264	0	69.0482	0	28.026	0.8057	18.0974	7.1298	3.6033	14.3203	1.0378 × 10^−14^
	Rank	1	10	1	7	1	9	5	12	8	6	11	4
F17	Avg	0	151.8179	0	109.6198	4.9084	194.1359	0.2559	216.455	30.7778	8.7194	197.9083	0
	Std	0	28.435	0	79.6068	26.8846	33.0475	0.4747	23.1197	3.1702	3.0756	19.4055	0
	Rank	1	9	1	8	5	10	4	12	7	6	11	1
F18	Avg	4.4409 × 10^−16^	2.5742	4.4409 × 10**^−16^**	2.7789 × 10^−8^	3.1678 × 10^−15^	8.1913	4.4409 × 10^−16^	9.3502	1.2613 × 10^−2^	0.8511	2.3255	3.6415 × 10^−15^
	Std	0	0.4745	0	2.7043 × 10^−9^	2.5861 × 10^−15^	7.6507	0	0.946	3.3014 × 10^−3^	0.6142	0.4014	2.1580 × 10^−15^
	Rank	1	10	1	6	4	11	1	12	7	8	9	5
F19	Avg	0	0.1297	8.3647 × 10^−31^	1.0441 × 10^−11^	9.3855 × 10^−3^	240.6283	0	51.1922	5.7896 × 10^−2^	0.1998	1.2171	5.3087 × 10^−2^
	Std	0	5.8069 × 10^−02^	9.1238 × 10^−30^	8.8748 × 10^−12^	3.6288 × 10^−2^	219.7319	0	16.6066	5.7987 × 10^−2^	0.2448	9.1563 × 10^−2^	0.2087
	Rank	1	8	3	4	5	12	1	11	7	9	10	6
F20	Avg	0	5.5514	0	2.9520 × 10^−9^	1.0727 × 10^−44^	6.33	4.9382 × 10^−147^	25.9518	3.0941 × 10^−2^	1.1060 × 10^−3^	10.4077	2.4419 × 10^−62^
	Std	0	2.3383	0	8.8910 × 10^−9^	5.8751 × 10^−44^	2.6195	6.0974 × 10^−147^	4.7219	1.0909 × 10^−2^	1.6310 × 10^−3^	2.5345	6.5463 × 10^−62^
	Rank	1	9	1	6	5	10	3	12	8	7	11	4
F21	Avg	2.5547 × 10^−2^	5.2594 × 10^−2^	2.9661 × 10^−2^	0.5326	1.9728 × 10^−2^	3.8312	2.2408 × 10^−3^	3.2529	4.4463 × 10^−6^	2.0755 × 10^−2^	0.0312	3.5037 × 10^−2^
	Std	9.7752 × 10^−3^	4.9296 × 10^−2^	1.4688 × 10^−2^	0.1176	5.4402 × 10^−2^	1.3479	1.6259 × 10^−3^	1.2921	4.5629 × 10^−6^	4.2171 × 10^−2^	2.6494 × 10^−2^	0.03
	Rank	5	9	4	10	3	12	2	11	1	4	6	7
F22	Avg	2.9038	0.5688	2.9005	2.7472	0.1729	9.1851 × 10^−3^	1.0042	2.7355	4.4222 × 10^−6^	5.5565 × 10^−3^	4.5368 × 10^−2^	0.134
	Std	0.1621	0.247	0.19955	0.3013	0.1276	6.9460 × 10^−3^	0.8045	0.8778	5.7875 × 10^−6^	1.0655 × 10^−2^	1.8046 × 10^−2^	0.0886
	Rank	12	7	9	10	6	3	8	9	1	2	4	5
F23	Avg	0.5537	6.2603	1.3854	12.1723	0.3206	0.8029	0.7788	5.8924	3.9947 × 10^−2^	6.3801 × 10^−2^	1.6454	0.5537
	Std	1.1402	3.5956	0.8307	2.4477	0.2835	0.6338	0.8349	1.7669	0.012	8.4765 × 10^−2^	0.6333	1.1402
	Rank	4	11	5	12	3	6	5	10	1	2	9	4
F24	Avg	0	3.7947	8.1486 × 10^−8^	1.0973	0	9.6985	0	38.4245	0	1.4924 × 10^−5^	0	0
	Std	0	3.1278	8.6874 × 10^−8^	2.0728	0	6.5506	0	2.5571	0	5.6480 × 10^−5^	0	0
	Rank	1	10	7	9	3	11	4	12	5	8	6	7
F25	Avg	0	1.6918	0	0.714	0.1592	1.844	0.0233	20.7438	0.732	4.9581	1.0114	0.136
	Std	0	0.4571	0	0.2408	0.1373	0.4942	4.2871 × 10^−2^	6.4717	0.2354	1.5861	0.2408	0.1394
	Rank	1	9	1	6	5	10	3	12	7	11	8	4
F26	Avg	0	22.3883	0	7.8537 × 10^−11^	0	4.9249	0	58.728	1.6200 × 10^−2^	0.4135	4.5409	0
	Std	0	6.0981	0	8.3251 × 10^−12^	0	2.077	0	12.0427	8.5765 × 10^−2^	1.0675	1.4705	0
	Rank	1	11	1	6	1	10	1	12	7	8	9	1
Paired rank +/=/−			24/0/2	11/12/3	25/0/1	18/3/5	23/0/3	19/3/4	24/0/2	21/0/5	18/3/5	24/0/2	20/4/2
Avg. rank		**2.26**	9.27	2.92	7.35	4.54	8.92	4.31	11.35	5.96	6.08	8.42	4.81
Overall rank		**1**	11	2	8	4	10	3	12	6	7	9	5

**Note:** The optimal values are highlighted in bold.

**Table 4:** A comparison of the best fitness values between IVYPSO and 10 other algorithms for various test functions.

Func.	Metrics	IVYPSO	PSO	IVY	BOA	WOA	GOOSE	HPSOBOA	dFDB_LSHADE	FDC_AGDE	NSM_BO	dFDB_SFS	FDB_AGSK
F1	Best	0	1.9195	3.2648 × 10^−260^	7.6243 × 10^−11^	2.3137 × 10^−83^	0.0134	2.2327 × 10^−291^	6221.6016	0.0075	3.9426 × 10^−5^	26.8336	3.2275 × 10^−104^
	Rank	1	10	3	6	5	9	2	12	8	7	11	4
F2	Best	0	4.8081	0	2.4927 × 10^−08^	3.6914 × 10^−49^	12.8456	7.8194 × 10^−146^	32.7774	0.0182	0.0051	5.3565	5.2776 × 10^−63^
	Rank	1	9	1	6	5	11	3	12	8	7	10	4
F3	Best	0	53.1933	6.5785 × 10^−95^	5.085 × 10^−11^	428.7845	0.7136	2.9839 × 10^−292^	169.5741	4.42	1.3085	161.3353	724.5566
	Rank	1	8	3	4	11	5	2	10	7	6	9	12
F4	Best	0	1.8277	0	2.466 × 10^−08^	0.4163	0.3557	5.2086 × 10^−147^	2.9046	1.4183	2.6106	1.0204	8.0432
	Rank	1	9	1	4	6	5	3	11	8	10	7	12
F5	Best	0.2523	0.9559	0.6904	5.1787	0.0725	0.007	0.0302	39.4716	5.2662 × 10^−5^	3.4297 × 10^−5^	0.2432	0.136
	Rank	8	10	9	11	5	3	4	12	2	1	7	6
F6	Best	1.1454 × 10^−5^	45.8573	3.2954 × 10**^−5^**	0.0014	0.0011	0.1351	5.385 × 10^−5^	2.6236	0.066	0.1496	0.0931	1.2591 × 10^−5^
	Rank	2	12	1	6	5	9	4	11	7	10	8	3
F7	Best	3.1914 × 10^−19^	0	6.3819 × 10^−35^	5.3719 × 10^−13^	7.1751 × 10^−66^	9.334 × 10^−66^	1.7703 × 10^−62^	8.2727 × 10^−52^	7.1751 × 10^−66^	7.1751 × 10^−66^	1.1911 × 10^−65^	7.1751 × 10^−66^
	Rank	11	1	10	12	2	6	8	9	2	2	7	2
F8	Best	0	0.3335	0	1.1492 × 10^−13^	2.5197 × 10^−100^	8.3454 × 10^−6^	1.7958 × 10^−295^	1.4189 × 10^−5^	4.3298 × 10^−21^	1.2446 × 10^−23^	9.3771 × 10^−11^	7.2831 × 10^−152^
	Rank	1	12	1	8	5	10	3	11	7	6	9	4
F9	Best	0	25.8431	4.3686 × 10^−95^	7.5806 × 10^−11^	1.2833 × 10^−82^	0.7984	2.2617 × 10^−291^	545.8044	0.0003	0.0003	4.3525	2.3672 × 10^−106^
	Rank	1	11	4	6	5	9	2	12	8	7	10	3
F10	Best	27.0059	673.4298	27.899	28.9468	27.7308	30.1997	28.8365	21,955.7713	29.2534	396.3681	203.3141	28.7384
	Rank	1	11	3	6	2	8	5	12	7	10	9	4
F11	Best	0	85.9941	0	6.8182 × 10^−11^	3.5927 × 10^−81^	0.244	1.9516 × 10^−291^	894.8034	0.0007	1.665 × 10^−5^	0.9829	1.1737 × 10^−108^
	Rank	1	11	1	6	5	9	3	12	8	7	10	4
F12	Best	0.6667	222.886	0.66667	0.9632	0.6667	0.8154	0.9953	2331.6115	0.7225	6.9031	4.5096	0.6774
	Rank	1	11	1	7	1	6	8	12	5	10	9	4
F13	Best	0	2.0826 × 10^−27^	0	5.5035 × 10^−26^	0	0.0017	0.0598	0.0089	4.8456 × 10^−42^	0	2.4964 × 10^−33^	0
	Rank	1	8	1	9	1	10	12	11	6	1	7	1
F14	Best	0	7.0078 × 10^−22^	0	7.8597 × 10^−20^	5.0035 × 10^−125^	0.013	0.0256	0.5158	5.1081 × 10^−27^	0	1.1492 × 10^−19^	1.0275 × 10^−152^
	Rank	1	7	1	8	5	10	11	12	6	1	9	4
F15	Best	0	1.1273 × 10^−30^	8.3154 × 10^−262^	5.7109 × 10^−22^	4.1221 × 10^−133^	0.0004	1.9605 × 10^−296^	0.0002	2.3713 × 10^−40^	0	2.0643 × 10^−31^	1.7744 × 10^−188^
	Rank	1	9	4	10	6	12	3	11	7	1	8	5
F16	Best	0	207.2783	0	2.8422 × 10^−13^	1.1369 × 10^−13^	170.5284	0	227.4552	45.5111	6.967	197.8528	0
	Rank	1	11	1	6	5	9	1	12	8	7	10	1
F17	Best	0	174.921	0	167.5903	0	280.0004	0	217.8745	35.3575	6.0006	206.6572	0
	Rank	1	9	1	8	1	12	1	11	7	6	10	1
F18	Best	4.4409 × 10^−16^	2.8075	4.4409 × 10**^−16^**	2.5252 × 10^−8^	4.4409 × 10^−16^	0.0649	4.4409 × 10^−16^	9.5098	0.0115	0.9373	2.0932	3.9968 × 10^−15^
	Rank	1	11	1	6	1	8	1	12	7	9	10	5
F19	Best	0	0.1039	0	1.8527 × 10^−11^	0	289.0103	0	57.1856	0.0152	0.1684	1.1599	0
	Rank	1	8	1	6	1	12	1	11	7	9	10	1
F20	Best	0	2.3272	0	5.0467 × 10^−10^	2.1642 × 10^−50^	8.4344	6.1344 × 10^−147^	23.0176	0.0272	0.0005	8.9652	1.3616 × 10^−67^
	Rank	1	9	1	6	5	10	3	12	8	7	11	4
F21	Best	0.0068	0.0125	0.0198	0.4861	0.0181	3.3439	0.0013	3.2316	4.0407 × 10^−6^	2.7412 × 10^−7^	0.0211	0.0107
	Rank	4	6	8	10	7	12	3	11	2	1	9	5
F22	Best	2.9661	0.8753	2.9715	2.9968	0.0595	0.0147	0.0651	1.5015	2.0754 × 10^−6^	7.3649 × 10^−9^	0.052	0.2135
	Rank	10	8	11	12	5	3	6	9	2	1	4	7
F23	Best	0.0134	5.2542	2.1048	6.4945	0.3179	0.2986	1.5223	5.6278	0.051	1.235	1.6193	0.0021
	Rank	2	10	9	12	5	4	7	11	3	6	8	1
F24	Best	0	3.2678	0	0	0	2.0776	0	38.2506	0	0	0	0
	Rank	1	11	1	1	1	10	1	12	1	1	1	1
F25	Best	0	2.4874	0	0.8955	0.398	1.5919	0.0995	25.468	0.8955	6.3676	0.9069	0.0995
	Rank	1	10	1	7	5	9	4	12	6	11	8	3
F26	Best	0	14.0908	0	7.6919 × 10^−11^	0	5.3631	0	54.1892	0.0006	0.0253	3.8484	0
	Rank	1	11	1	6	1	10	1	12	7	8	9	1
Paired rank +/=/−			24/0/2	9/15/2	25/0/1	16/7/3	23/0/3	16/6/4	24/0/2	21/1/4	18/4/4	22/1/3	16/6/4
Avg. rank		**2.19**	9.35	3.08	7.27	4.08	8.5	3.92	11.35	5.92	5.85	8.46	3.92
Overall rank		**1**	10	2	8	5	10	3	11	7	6	9	3

**Note:** The optimal values are highlighted in bold.

**Table 5:** Results of the Wilcoxon signed-rank test for 26 test functions with α = 0.05.

Algorthm	Wilcoxon Test *p*-Value	Significant
IVYPSO-PSO	2.4153 × 10^−5^	Yes
IVYPSO-IVY	1.6357 × 10^−2^	Yes
IVYPSO-BOA	2.9991 × 10^−5^	Yes
IVYPSO-WOA	4.5685 × 10^−2^	Yes
IVYPSO-GOOSE	5.0978 × 10^−5^	Yes
IVYPSO-HPSOBOA	1.6067 × 10^−2^	Yes
IVYPSO-FDC-AGDE	8.3166 × 10^−3^	Yes
IVYPSO-dFDB-LSHADE	7.8847 × 10^−6^	Yes
IVYPSO-NSM-BO	3.8919 × 10^−3^	Yes
IVYPSO-dFDB-SFS	5.9619 × 10^−5^	Yes
IVYPSO-FDB-AGSK	1.5664 × 10^−3^	Yes

**Table 6:** Friedman ranking scores of IVYPSO and other competing algorithms.

Algorthm	Friedman Scores	Rank
**IVYPSO**	**1.9231**	**1**
PSO	5.8846	9
IVY	3.7308	2
BOA	6.7308	11
WOA	4.8462	3
GOOSE	5.4615	6
HPSOBOA	4.9615	4
FDC-AGDE	7.0385	12
dFDB-LSHADE	6.4231	10
NSM-BO	5.4615	7
dFDB-SFS	5.5385	8
FDB-AGSK	5.0385	5

**Note:** The optimal values are highlighted in bold.

**Table 7:** The best values obtained by IVYPSO and other competing algorithms for the GTCD problem.

Algorithm	L	r	D	Optimal Value	SR (%)	ACTs	AFEs
IVYPSO	24.496	1.5867	20	**1,677,759.2755**	**100**	0.1419	15,030
PSO	24.496	1.5867	20	**1,677,759.2755**	80	0.1187	15,030
IVY	24.496	1.5867	20	**1,677,759.2755**	80	0.172	15,030
BOA	20	1.1134	20	1,683,684.5457	0	0.1797	30,030
WOA	24.496	1.5867	20	**1,677,759.2755**	85	0.0938	15,000
GOOSE	32.5256	1.2305	20	1,677,759.2854	0	0.101	15,000
HPSOBOA	21	1.0537	21	1,685,732.6804	0	0.1786	30,030
FDC**_**AGDE	24.496	1.5867	20	**1,677,759.2755**	85	0.1087	15,030
dFDB**_**LSHADE	28.5541	1.1932	20	1,677,783.3132	0	**0.0047**	500
NSM**_**BO	24.496	1.5867	20	**1,677,759.2755**	**100**	0.3239	15,000
dFDB**_**SFS	24.496	1.5867	20	**1,677,759.2755**	80	0.1978	15,000
FDB**_**AGSK	24.496	1.5867	20	**1,677,759.2755**	**100**	0.1845	15,000

**Note:** The optimal values are highlighted in bold.

**Table 8:** Statistical assessment of various algorithms applied to the GTCD problem.

Algorithm	Mean	Best	Worst	Median	Std	Rank
IVYPSO	**1,677,759.2755**	1,677,759.2755	1,677,759.2755	1,677,759.2755	0.0000	**1**
PSO	1,678,556.6157	1,677,759.2755	1,685,732.6774	1,677,759.2755	2521.4111	7
IVY	1,678,354.4837	1,677,759.2755	1,685,634.2755	1,677,759.2755	1932.6400	6
BOA	1,685,527.8813	1,683,684.5457	1,685,732.7254	1,685,732.6942	647.6821	8
WOA	1,677,759.2760	1,677,759.2755	1,677,759.2777	1,677,759.2756	0.0009	4
GOOSE	2,048,858.2698	1,677,759.2854	5,177,439.6140	1,698,815.7980	1,099,516.1908	12
HPSOBOA	1,685,748.4393	1,685,732.6804	1,685,810.0629	1,685,735.5707	25.3056	9
FDC**_**AGDE	1,778,373.1947	1,677,759.2755	2,675,925.0653	1,677,759.2755	315,377.5360	11
dFDB**_**LSHADE	1,678,255.5734	1,677,783.3132	1,679,765.5234	1,678,075.9952	619.2305	5
NSM**_**BO	**1,677,759.2755**	1,677,759.2755	1,677,759.2755	1,677,759.2755	0	**1**
dFDB**_**SFS	1,694,760.8055	1,677,759.2755	1,762,766.9254	1,677,759.2755	35,842.3723	10
FDB**_**AGSK	**1,677,759.2755**	1,677,759.2755	1,677,759.2755	1,677,759.2755	0	**1**

**Note:** The optimal values are highlighted in bold.

**Table 9:** The best values obtained by IVYPSO and other competing algorithms for the three-bar truss design problem.

Algorithm	X1	X2	Optimal Value	SR (%)	ACTs	AFEs
IVYPSO	0.7884	0.4081	**263.8523**	**100**	0.1531	15,030
PSO	0.7884	0.4081	**263.8523**	85	0.1663	15,030
IVY	0.7884	0.4081	**263.8523**	90	0.2151	15,030
BOA	0.7937	0.3938	263.8849	0	0.2743	30,030
WOA	0.7919	0.3984	263.8611	0	0.137	15,000
GOOSE	0.7884	0.4081	**263.8523**	**100**	0.1447	15,000
HPSOBOA	0.8374	0.4669	264.2474	0	0.2783	30,030
FDC**_**AGDE	0.7884	0.4081	**263.8523**	**100**	0.1518	15,030
dFDB**_**LSHADE	0.7884	0.4081	**263.8523**	90	**0.0058**	500
NSM**_**BO	0.7884	0.4081	**263.8523**	**100**	0.357	15,000
dFDB**_**SFS	0.7884	0.4081	**263.8523**	**100**	0.2654	15,000
FDB**_**AGSK	0.7884	0.4081	**263.8523**	**100**	0.2167	15,000

**Note:** The optimal values are highlighted in bold.

**Table 10:** Statistical assessment of various algorithms applied to the three-bar truss design problem.

Algorithm	Mean	Best	Worst	Median	Std	Rank
IVYPSO	**263.8523**	263.8523	263.8523	263.8523	0	**1**
PSO	263.9375	263.8523	264.7016	263.8523	0.2685	9
IVY	263.8524	263.8523	263.8527	263.8524	6.97 × 10^−4^	7
BOA	264.1555	263.8787	264.8682	264.0228	0.3227	10
WOA	265.3408	263.8591	268.7184	264.7459	1.8316	11
GOOSE	263.8524	263.8523	264.5934	263.8523	0.2397	6
HPSOBOA	271.5462	264.4945	279.0356	272.9894	4.4763	12
FDC**_**AGDE	**263.8523**	263.8523	263.8523	263.8523	0	**1**
dFDB**_**LSHADE	263.8573	263.8524	263.8955	263.8527	0.0134	8
NSM**_**BO	**263.8523**	263.8523	263.8523	263.8523	0	**1**
dFDB**_**SFS	**263.8523**	263.8523	263.8523	263.8523	0	**1**
FDB**_**AGSK	**263.8523**	263.8523	263.8523	263.8523	0	**1**

**Note:** The optimal values are highlighted in bold.

**Table 11:** The best values obtained by IVYPSO and other competing algorithms for the multiple-disk clutch brake design problem.

Algorithm	X1	X2	X3	X4	X5	Optimal Value	SR(%)	ACTs	AFEs
IVYPSO	70	90	1	1000	2	**0.2352**	**100**	0.2261	15,030
PSO	70	90	1	1000	2	**0.2352**	90	0.2117	15,030
IVY	70	90	1	1000	2	**0.2352**	95	0.2644	15,030
BOA	69.7384	90	1.1944	405.0447	2	0.2842	0	0.3671	30,030
WOA	70	90	1	1000	2	**0.2352**	**100**	0.1843	15,000
GOOSE	70	90	1	1000	2	**0.2352**	90	0.2395	15,000
HPSOBOA	67.2176	91	0.8157	788.6999	1.8812	0.2731	0	0.6078	30,030
FDC**_**AGDE	70	90	1	1000	2	**0.2352**	**100**	0.2211	15,030
dFDB**_**LSHADE	69.9485	90	1	432.177	2	0.2358	0	**0.0117**	500
NSM**_**BO	70	90	1	1000	2	**0.2352**	**100**	0.4497	15,000
dFDB**_**SFS	70	90	1	1000	2	**0.2352**	85	0.3248	15,000
FDB**_**AGSK	70	90	1	1000	2	**0.2352**	**100**	0.2867	15,000

**Note:** The optimal values are highlighted in bold.

**Table 12:** Statistical assessment of various algorithms applied to the multiple-disk clutch brake design problem.

Algorithm	Mean	Best	Worst	Median	Std	Rank
IVYPSO	**0.2352**	0.2352	0.2352	0.2352	0	**1**
PSO	0.2381	0.2352	0.2638	0.2352	0.009	8
IVY	0.2354	0.2352	0.236	0.2352	0.0014	6
BOA	0.3149	0.2842	0.3308	0.3255	0.0234	11
WOA	**0.2352**	0.2352	0.2352	0.2352	0	**1**
GOOSE	0.2383	0.2352	0.2531	0.2352	0.006	9
HPSOBOA	0.3264	0.2731	0.3308	0.3308	0.0139	12
FDC**_**AGDE	**0.2352**	0.2352	0.2352	0.2352	0	**1**
dFDB**_**LSHADE	0.2376	0.2358	0.242	0.2371	0.002	7
NSM**_**BO	**0.2352**	0.2352	0.2352	0.2352	0	**1**
dFDB**_**SFS	0.2411	0.2352	0.2646	0.2352	0.0124	10
FDB**_**AGSK	**0.2352**	0.2352	0.2352	0.2352	0	**1**

**Note:** The optimal values are highlighted in bold.

## Data Availability

The datasets used and/or analyzed during the current study are publicly available and can be accessed without any restrictions. If needed, they can also be obtained by contacting the corresponding author.

## References

[1] Sattar D., Salim R. (2021). A smart metaheuristic algorithm for solving engineering problems. Eng. Comput..

[2] Abualigah L., Elaziz M.A., Khasawneh A.M., Alshinwan M., Ibrahim R.A., Al-Qaness M.A.A., Mirjalili S., Sumari P., Gandomi A.H. (2022). Meta-heuristic optimization algorithms for solving real-world mechanical engineering design problems: A comprehensive survey, applications, comparative analysis, and results. Neural Comput. Appl..

[3] Talbi E.G. (2021). Machine learning into metaheuristics: A survey and taxonomy. ACM Comput. Surv. (CSUR).

[4] Kaveh M., Mesgari M.S. (2023). Application of meta-heuristic algorithms for training neural networks and deep learning architectures: A comprehensive review. Neural Process. Lett..

[5] Katebi J., Shoaei-Parchin M., Shariati M., Trung N.T., Khorami M. (2020). Developed comparative analysis of metaheuristic optimization algorithms for optimal active control of structures. Eng. Comput..

[6] Joseph S.B., Dada E.G., Abidemi A., Oyewola D.O., Khammas B.M. (2022). Metaheuristic algorithms for PID controller parameters tuning: Review, approaches and open problems. Heliyon.

[7] Ahmadianfar I., Bozorg-Haddad O., Chu X. (2020). Gradient-based optimizer: A new metaheuristic optimization algorithm. Inf. Sci..

[8] Abdel-Basset M., Mohamed R., Elkomy O.M., Abouhawwash M. (2022). Recent metaheuristic algorithms with genetic operators for high-dimensional knapsack instances: A comparative study. Comput. Ind. Eng..

[9] Keivanian F., Chiong R. (2022). A novel hybrid fuzzy–metaheuristic approach for multimodal single and multi-objective optimization problems. Expert Syst. Appl..

[10] Huang Y., Lu S., Liu Q., Han T., Li T. (2025). GOHBA: Improved Honey Badger Algorithm for Global Optimization. Biomimetics.

[11] Zhu X., Zhang J., Jia C., Liu Y., Fu M. (2025). A Hybrid Black-Winged Kite Algorithm with PSO and Differential Mutation for Superior Global Optimization and Engineering Applications. Biomimetics.

[12] Choi K., Jang D.-H., Kang S.-I., Lee J.-H., Chung T.-K., Kim H.-S. (2015). Hybrid algorithm combing genetic algorithm with evolution strategy for antenna design. IEEE Trans. Magn..

[13] Yu X., Jiang N., Wang X., Li M. (2023). A hybrid algorithm based on grey wolf optimizer and differential evolution for UAV path planning. Expert Syst. Appl..

[14] Peng Y., Wang Y., Hu F., He M., Mao Z., Huang X., Ding J. (2024). Predictive modeling of flexible EHD pumps using Kolmogorov–Arnold Networks. Biomim. Intell. Robot..

[15] Mao Z., Bai X., Peng Y., Shen Y. (2024). Design, modeling, and characteristics of ring-shaped robot actuated by functional fluid. J. Intell. Mater. Syst. Struct..

[16] Kennedy J., Eberhart R. (1995). Particle swarm optimization. Proceedings of the ICNN’95-International Conference on Neural Networks.

[17] Papazoglou G., Biskas P. (2023). Review and comparison of genetic algorithm and particle swarm optimization in the optimal power flow problem. Energies.

[18] Thangaraj R., Pant M., Abraham A., Bouvry P. (2011). Particle swarm optimization: Hybridization perspectives and experimental illustrations. Appl. Math. Comput..

[19] Sun J., Xu W., Feng B. (2005). Adaptive parameter control for quantum-behaved particle swarm optimization on individual level. Proceedings of the 2005 IEEE International Conference on Systems, Man and Cybernetics.

[20] Song Y., Liu Y., Chen H., Deng W. (2023). A multi-strategy adaptive particle swarm optimization algorithm for solving optimization problem. Electronics.

[21] Ghasemi M., Zare M., Trojovský P., Rao R.V., Trojovská E., Kandasamy V. (2024). Optimization based on the smart behavior of plants with its engineering applications: Ivy algorithm. Knowl. Based Syst..

[22] Kahraman H.T., Aras S., Gedikli E. (2020). Fitness-distance balance (FDB): A new selection method for meta-heuristic search algorithms. Knowl. Based Syst..

[23] Duman S., Kahraman H.T., Kati M. (2023). Economical operation of modern power grids incorporating uncertainties of renewable energy sources and load demand using the adaptive fitness-distance balance-based stochastic fractal search algorithm. Eng. Appl. Artif. Intell..

[24] Kahraman H.T., Bakir H., Duman S., Katı M., Aras S., Guvenc U. (2022). Dynamic FDB selection method and its application: Modeling and optimizing of directional overcurrent relays coordination. Appl. Intell..

[25] Ozkaya B., Kahraman H.T., Duman S., Guvenc U. (2023). Fitness-Distance-Constraint (FDC) based guide selection method for constrained optimization problems. Appl. Soft Comput..

[26] Yildirim I., Bozkurt M.H., Kahraman H.T., Aras S. (2025). Dental X-Ray image enhancement using a novel evolutionary optimization algorithm. Eng. Appl. Artif. Intell..

[27] Kahraman H.T., Hassan M.H., Katı M., Tostado-Véliz M., Duman S., Kamel S. (2024). Dynamic-fitness-distance-balance stochastic fractal search (dFDB-SFS algorithm): An effective metaheuristic for global optimization and accurate photovoltaic modeling. Soft Comput..

[28] Bakır H., Duman S., Guvenc U., Kahraman H.T. (2023). Improved adaptive gaining-sharing knowledge algorithm with FDB-based guiding mechanism for optimization of optimal reactive power flow problem. Electr. Eng..

[29] Hamad R.K., Rashid T.A. (2024). GOOSE algorithm: A powerful optimization tool for real-world engineering challenges and beyond. Evol. Syst..

[30] Zhang M., Long D., Qin T., Yang J. (2020). A chaotic hybrid butterfly optimization algorithm with particle swarm optimization for high-dimensional optimization problems. Symmetry.

[31] Kahraman H.T., Katı M., Aras S., Taşci D.A. (2023). Development of the Natural Survivor Method (NSM) for designing an updating mechanism in metaheuristic search algorithms. Eng. Appl. Artif. Intell..

[32] Öztürk H.T., Kahraman H.T. (2025). Metaheuristic search algorithms in Frequency Constrained Truss Problems: Four improved evolutionary algorithms, optimal solutions and stability analysis. Appl. Soft Comput..

[33] Ouyang H., Li W., Gao F., Huang K., Xiao P. (2024). Research on Fault Diagnosis of Ship Diesel Generator System Based on IVY-RF. Energies.

[34] Jamil M., Yang X.S. (2013). A literature survey of benchmark functions for global optimisation problems. Int. J. Math. Model. Numer. Optim..

[35] Zhan Z.-H., Shi L., Tan K.C., Zhang J. (2022). A survey on evolutionary computation for complex continuous optimization. Artif. Intell. Rev..

[36] Cai T., Zhang S., Ye Z., Zhou W., Wang M., He Q., Chen Z., Bai W. (2024). Cooperative metaheuristic algorithm for global optimization and engineering problems inspired by heterosis theory. Sci. Rep..

[37] Mao Z., Hosoya N., Maeda S. (2024). Flexible electrohydrodynamic fluid-driven valveless water pump via immiscible interface. Cyborg Bionic Syst..

[38] Lau S.L.H., Lim J., Chong E.K.P., Wang X. (2023). Single-pixel image reconstruction based on block compressive sensing and convolutional neural network. Int. J. Hydromechatronics.

[39] Verma H., Siruvuri S.V., Budarapu P.R. (2024). A machine learning-based image classification of silicon solar cells. Int. J. Hydromechatronics.

[40] Alawi O.A., Kamar H.M., Shawkat M.M., Al Ani M.M., Mohammed H.A., Homod R.Z., Wahid M.A. (2024). Artificial intelligence-based viscosity prediction of polyalphaolefin-boron nitride nanofluids. Int. J. Hydromechatro..

[41] Arora S., Singh S. (2019). Butterfly optimization algorithm: A novel approach for global optimization. Soft Comput..

[42] Mirjalili S., Lewis A. (2016). The whale optimization algorithm. Adv. Eng. Softw..

[43] Yuan F., Huang X., Zheng L., Wang L., Wang Y., Yan X., Gu S., Peng Y. (2025). The Evolution and Optimization Strategies of a PBFT Consensus Algorithm for Consortium Blockchains. Information.

[44] Zhang C., Chen J., Li J., Peng Y., Mao Z. (2023). Large language models for human–robot interaction: A review. Biomim. Intell. Robot..

[45] Peng Y., Yang X., Li D., Ma Z., Liu Z., Bai X., Mao Z. (2025). Predicting flow status of a flexible rectifier using cognitive computing. Expert Syst. Appl..

[46] Bai X., Peng Y., Li D., Liu Z., Mao Z. (2024). Novel soft robotic finger model driven by electrohydrodynamic (EHD) pump. J. Zhejiang Univ. Sci. A.

[47] Mao Z., Peng Y., Hu C., Ding R., Yamada Y., Maeda S. (2023). Soft computing-based predictive modeling of flexible electrohydrodynamic pumps. Biomim. Intell. Robot..

[48] Peng Y., Sakai Y., Funabora Y., Yokoe K., Aoyama T., Doki S. (2025). Funabot-Sleeve: A Wearable Device Employing McKibben Artificial Muscles for Haptic Sensation in the Forearm. IEEE Robot. Autom. Lett..

[49] Mao Z., Kobayashi R., Nabae H., Suzumori K. (2024). Multimodal strain sensing system for shape recognition of tensegrity structures by combining traditional regression and deep learning approaches. IEEE Robot. Autom. Lett..

[50] Dai L., Zhang L., Chen Z. (2022). GrS Algorithm for Solving Gas Transmission Compressor Design Problem. Proceedings of the 2022 IEEE/WIC/ACM International Joint Conference on Web Intelligence and Intelligent Agent Technology (WI-IAT).

[51] Dhiman G., Kaur A. (2017). Spotted hyena optimizer for solving engineering design problems. Proceedings of the 2017 International Conference on Machine Learning and Data Science (MLDS).

